# Deficiency of mDia, an Actin Nucleator, Disrupts Integrity of Neuroepithelium and Causes Periventricular Dysplasia

**DOI:** 10.1371/journal.pone.0025465

**Published:** 2011-09-28

**Authors:** Dean Thumkeo, Ryota Shinohara, Keisuke Watanabe, Hirohide Takebayashi, Yosuke Toyoda, Kiyoshi Tohyama, Toshimasa Ishizaki, Tomoyuki Furuyashiki, Shuh Narumiya

**Affiliations:** 1 Department of Pharmacology, Kyoto University Faculty of Medicine, Kyoto, Japan; 2 Department of Morphological Neural Science, Graduate School of Medical Sciences, Kumamoto University, Kumamoto, Japan; Northwestern University Feinberg School of Medicine, United States of America

## Abstract

During development of the central nervous system, the apical-basal polarity of neuroepithelial cells is critical for homeostasis of proliferation and differentiation of neural stem cells. While adherens junctions at the apical surface of neuroepithelial cells are important for maintaining the polarity, the molecular mechanism regulating integrity of these adherens junctions remains largely unknown. Given the importance of actin cytoskeleton in adherens junctions, we have analyzed the role of mDia, an actin nucleator and a Rho effector, in the integrity of the apical adherens junction. Here we show that mDia1 and mDia3 are expressed in the developing brain, and that mDia3 is concentrated in the apical surface of neuroepithelium. Mice deficient in both mDia1 and mDia3 develop periventricular dysplastic mass widespread throughout the developing brain, where neuroepithelial cell polarity is impaired with attenuated apical actin belts and loss of apical adherens junctions. In addition, electron microscopic analysis revealed abnormal shrinkage and apical membrane bulging of neuroepithelial cells in the remaining areas. Furthermore, perturbation of Rho, but not that of ROCK, causes loss of the apical actin belt and adherens junctions similarly to mDia-deficient mice. These results suggest that actin cytoskeleton regulated by Rho-mDia pathway is critical for the integrity of the adherens junctions and the polarity of neuroepithelial cells, and that loss of this signaling induces aberrant, ectopic proliferation and differentiation of neural stem cells.

## Introduction

Neuroepithelial cells function as neural stem cells, which proliferate for self-renewal and to produce progenitor cells for lineages of cells in the central nervous system (CNS). After several rounds of cell division, the progenitor cells become differentiated to precursors of neurons or glial cells, which start to migrate towards the destination of final differentiation [1]. Neural stem cell division and differentiation thus need to be tightly controlled spatiotemporally. Neuroepithelial cells have the apical and the basal processes, and, most significantly, the cell-cell adhesion structure called the apical adherens junction which exists at the end feet of the apical process [2]. Apical adherens junctions are indispensable for the formation and maintenance of the apical-basal polarity of neuroepithelium [3]. Many studies in mouse and human genetics have identified importance of this polarity in homeostatic control of neural stem cells [4], [5], which functions together with differentiation machineries such as Notch and Sonic-Hedgehog signaling [5], [6]. Adherens junction is composed of the cadherin-catenin complex. Gene deletion of components of the adherens junction of neuroepithelial cells such as αE-catenin [6] and N-cadherin [7] perturbs their apical-basal polarity, releases neural stem cells from tight control to proliferate and differentiate autonomously, and induces formation of abnormal cellular mass. Since αE-catenin and N-cadherin function not only in apical adherens junctions but also in adherens junctions at other sites, mass can grow in any direction and invariably protrudes into the ventricle. Occurrence of such periventricular dysplastic mass demonstrates the importance of the apical adherens junction in maintenance of the neuroepithelial integrity. Histological analysis previously identified an actin belt linking apical adherens junctions and lining the apical surface of neuroepithelial cells. However, whether and how this apical actin belt is formed, maintained and supports the integrity of the apical adherens junctions of neuroepithelial cells remain poorly understood.

The Rho family of small GTPases is a major regulator of actin cytoskeleton in various cell types including neuronal cells [8]. Rho, Rac and Cdc42 are primary members of the Rho family of small GTPases, and each of these proteins mediates distinct types of actin reorganization in cultured cells [9]. Previous reports have suggested the role of Cdc42 in formation of the adherens junction of neuroepithelial cells [10], [11]. In one of these reports, Cdc42 deficiency leads to a gradual loss of the Par complex, a Cdc42 effector for cell polarity, from the apical surface of neuroepithelial cells and impairs adherens junction [10]. On the other hand, the role of Rho in regulating actin dynamics and apical adherens junction of the neuroepithelial cells remains to be elucidated. Rho regulates actomyosin-based contractility through binding to its effectors, ROCK and mDia [12]. Upon Rho activation, ROCK phosphorylates and activates myosin light chain to form actomyosin bundles [13], and mDia is critical for *de novo* actin filament formation [14]. Activated mDia nucleates actin oligomers and binds to the barbed end to produce long, straight actin filaments [15], [16]. In this study, we show that two mDia isoforms, mDia1 and mDia3, are expressed in the developing brain and that mDia3 localizes in the actin belt and adherens junction in the apical surface of neuroepithelial cells. To elucidate the mDia function in the apical adherens junction and the apical-basal polarity of neuroepithelial cells, we have generated mice lacking mDia1 and mDia3 in combination. We have found that the mDia deficiency induces loss of neuroepithelial apical adherens junction and polarity at multiple regions of the brain that eventually results in periventricular dysplastic mass. As development proceeds, periventricular dysplastic mass enlarges, occupies the ventricular space, and apparently obstructs the cerebrospinal fluid (CSF) circulation to cause hydrocephalus in these mice.

## Results

### Formation of periventricular dysplastic mass and hydrocephalus in mDia-deficient mice

**Figure 1:**
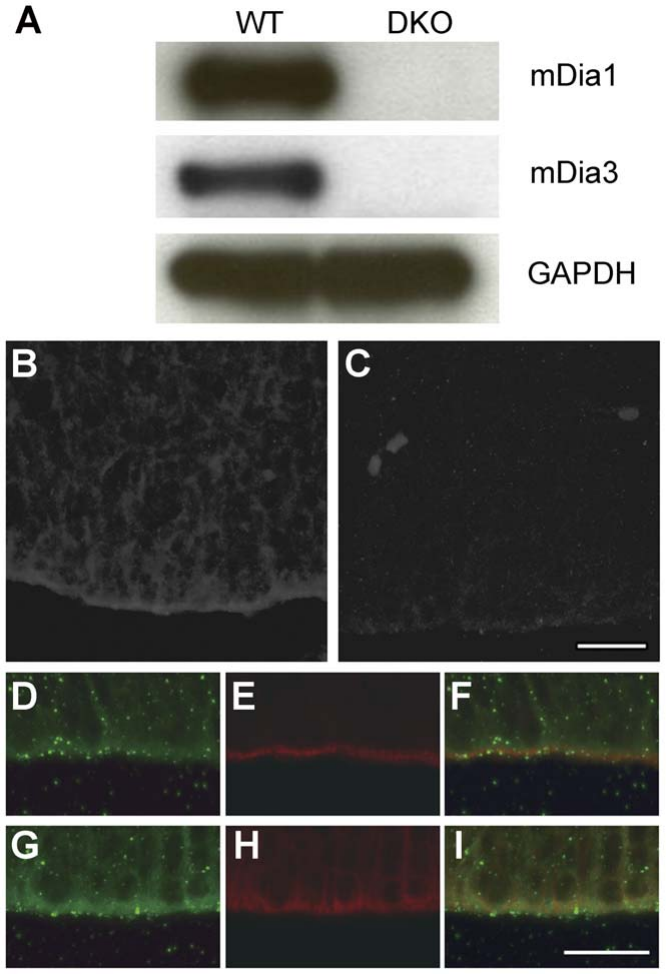
Expression of mDia1 and mDia3 in embryonic mouse forebrain. (A) Western blotting for protein expression of mDia1 and mDia3 in the mouse forebrain at embryonic day 16 (E16). The specificity of mDia1 and mDia3 signals was confirmed using lysates from mDia-DKO mice. GAPDH was used as an internal control. (B, C) Immunofluorescent staining for mDia3 in coronal brain sections from wild-type (B) and mDia3^null^ (C) embryos at E16. Note that mDia3 signals were enriched at the apical surface of the cerebral cortex in wild-type mice. (D–F) Immunofluorescent staining for mDia3 (green, D) and phalloidin staining (red, E) in coronal sections of the lateral ventrical wall from wild-type mice at E13. A merged image is shown in F. mDia3 accumulated at the apical surface and colocalized with filamentous actin. (G–I) Double immunofluorescent staining for mDia3 (green, G) and β-catenin (red, I) in coronal sections of the cerebral cortex from wild-type mice at E13. A merged image is shown in I. mDia3 also colocalized with β-catenin at the apical surface. (B, C) Scale bar, 20 µm. (D–I) Scale bar, 20 µm.

To investigate the role of mDia in formation and maintenance of brain architecture, we first examined expression of mDia isoforms in the developing brain. Western blotting using respective antibodies detected bands for mDia1 and mDia3 in the forebrain of E16 wild-type embryos (Fig. 1A). These signals were abolished in mice lacking mDia1 and mDia3 in combination, confirming the specificity of these signals (Fig. 1A). Immunofluorescent staining showed that signals for mDia3 are present in neuroepithelial cells and concentrated on the apical surface of E16 wild-type embryos (Fig. 1B). These mDia3 signals were abolished in mDia3-deficient mice (Fig. 1C), again confirming the specificity of these signals. Notably, these mDia3 signals are colocalized with signals for actin filaments (Fig. 1D, 1E, 1F) and β-catenin (Fig. 1G, 1H, 1I), suggesting that mDia3 localizes in the adherens junction and linking actin belt on the apical surface of neuroepithelial cells.

**Figure 2:**
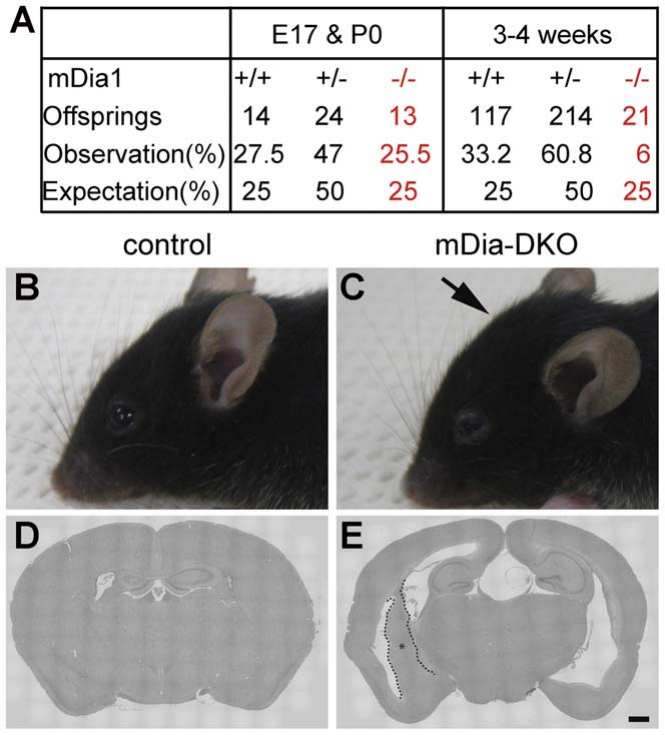
mDia-DKO mice develop hydrocephalus. (A) The proportion of mDia1 wild-type (+/+), heterozygous (+/−) and homozygous (−/−) mDia3^null^ offspring at around birth (E17 and P0) and weaning (3–4 weeks after birth) periods obtained by crossing mDia1^+/−^;mDia3^−/y^ male and mDia1^+/−^;mDia3^−/−^ female mice. Expected proportions of respective genotypes based on Mendelian frequency are also shown at the bottom. (B, C) Lateral view of the heads of control (B) and mDia-DKO mouse (C) of 3 months old. An arrow indicates a dome-like appearance of the head of mDia-DKO mouse. (D, E) H&E-staining coronal brain section of control (D) and mDia-DKO (E) mice. Note that the dorsal third ventricle and the lateral ventricle were markedly enlarged in mDia-DKO mouse. An asterisk indicates a region of periventricular dysplastic mass outlined by dotted lines. (D, E) Scale bar, 250 µm.

To address a role of mDia in neuroepithelial cells, we have generated mice deficient in either of mDia isoforms. As previously reported, mDia1-deficient (mDia1^−/−^) mice exhibit impaired T-cell trafficking, but otherwise are born and develop apparently normally [17]. mDia3^null^ mice are also born at the Mendelian expectation, develop without apparent abnormality and are fertile [Shinohara *et al.* submitted]. To examine possible redundancy among mDia isoforms, we have produced double knockout mice deficient in both mDia1 and mDia3 (mDia-DKO) by cross-mating mDia1^+/−^;mDia3^−/−^ females with mDia1^+/−^;mDia3^−/Y^ males. Analysis of the genotypes in offspring revealed that the number of mDia-DKO mice was as predicted by Mendelian inheritance around birth but significantly underrepresented in weaning period (Fig. 2A). By visual inspection, a signification portion (5 out of 11) of adult mDia-DKO mice displayed the head of a dome-like appearance (Fig. 2C) compared with the appearance of their control littermates (Fig. 2B). Histological analysis of coronal sections revealed various degrees of the dilation of the lateral and the third ventricle in all adult mDia-DKO mice analyzed (n = 11) (Fig. 2D, 2E), revealing the presence of hydrocephalus in mDia-DKO mice. Notably, we found a large mass of periventricular dysplasia in the third ventricle (Fig. S1A and S1B), which possibly obstructed CSF circulation and dilated the ventricles due to increased hydrostatic pressure. A similar periventricular dysplastic mass was also noted in the lateral ventricle (Fig. 2E, Fig. S1C and S1D).

### Loss of mDia affects the apical actin belt, the apical adherens junction and the apical-basal polarity of neuroepithelial cells during embryonic brain development

**Figure 3:**
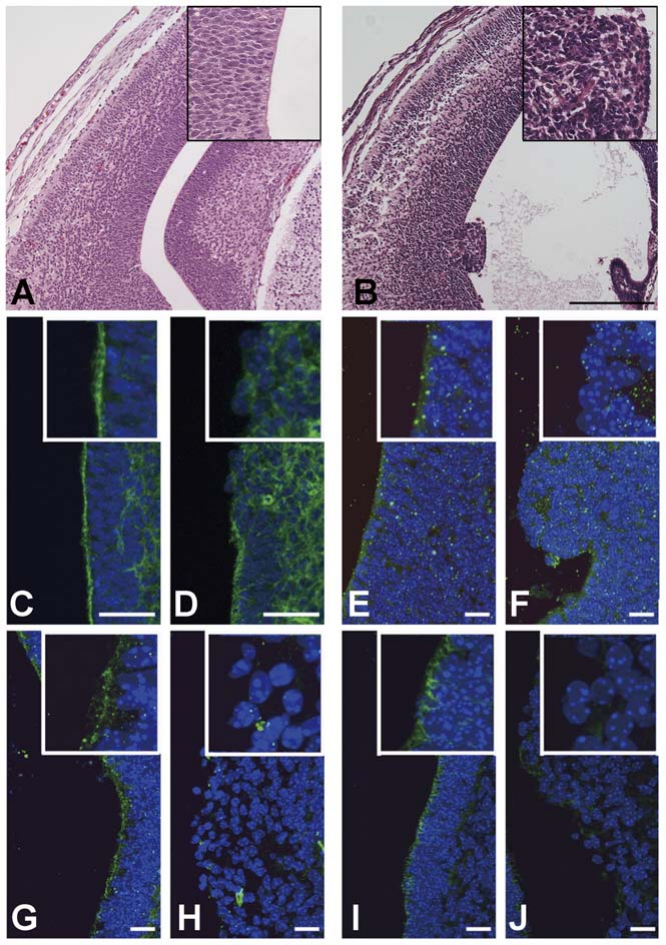
Widespread disruption of structural integrity and apical-basal polarity of neuroepithelium in developing brain of mDia-DKO mice. (A, B) H&E-stained coronal sections of the lateral ventricle wall from control (A) and mDia-DKO (B) mice at E14. Insets show higher magnification of H&E staining of A and B. In mDia-DKO mice, the lateral ventricle was enlarged and the neuroepithelial cell polarity was lost. periventricular dysplastic mass protruded into the lateral ventricle was also observed. (C, D) Phalloidin staining (green) in coronal sections of the third ventricular wall from control (C) and mDia-DKO mice (D) at E14. Note that disruption of actin filament belt lining the ventricle wall was observed in mDia-DKO mice. (E, F) Immunofluorescent staining for N-cadherin (green) in coronal sections of the lateral ventricular wall from control (E) and mDia-DKO (F) mice at E14. (G, H) Immunofluorescent staining for aPKCλ (green) in coronal sections of the third ventricular wall from control (G) and mDia-DKO (H) mice at E14. (I, J) Immunofluorescent staining for β-catenin (green) in coronal sections of the third ventricular wall from control (I) and mDia-DKO (J) mice at E14. Nuclei were counterstained by Hoechst (blue). Insets show higher magnification. (A, B) Scale bar, 250 µm, (C, D) Scale bars, 50 µm. (E–J) Scale bars, 25 µm.

To figure out how periventricular dysplastic mass is formed in mDia-DKO mice, we histologically analyzed the brain of this genotype of mice during early development. A coronal section of the lateral ventricle with hematoxylin and eosin (H&E) staining shows abnormal alignment and denudation of a part of neuroepithelial cells lining the lateral ventricle at E14 (Fig. 3A, 3B). Similar abnormalities were also observed in the third ventricle, the aqueduct, the forth ventricle (Fig. S2A, S2B, S2C, S2D, S2E, S2F) and the central canal of spinal cord (data not shown), indicating impaired integrity of the neuroepithelium at multiple regions throughout the brain. The apical integrity of the neuroepithelium is maintained by the apical adherens junction and the actin belt linking these junctions. Given that mDia is an actin nucleator [14], we then visualized filamentous actin structures in neuroepithelial cells by fluorescently labeled phalloidin. Whereas actin filaments continuously lined the apical surface of neuroepithelial cells along the ventricle wall as a belt in E14 wild-type embryos, such an actin belt was often disrupted in its continuity and disappeared in regions where neuroepithelial cells were misaligned and denuded in mDia-DKO mice (Fig. 3C, 3D). Concomitant with the loss of apical actin belt, the localization to the apical surface of components of the adherens junction such as N-cadherin (Fig. 3E, 3F), aPKCλ (Fig. 3G, 3H) and β-catenin (Fig. 3I, 3J) were impaired in these regions of mDia-DKO mice. This deficit was not due to altered expression or stability of these proteins, because the mDia deficiency did not alter their protein levels (Fig. S3).

**Figure 4:**
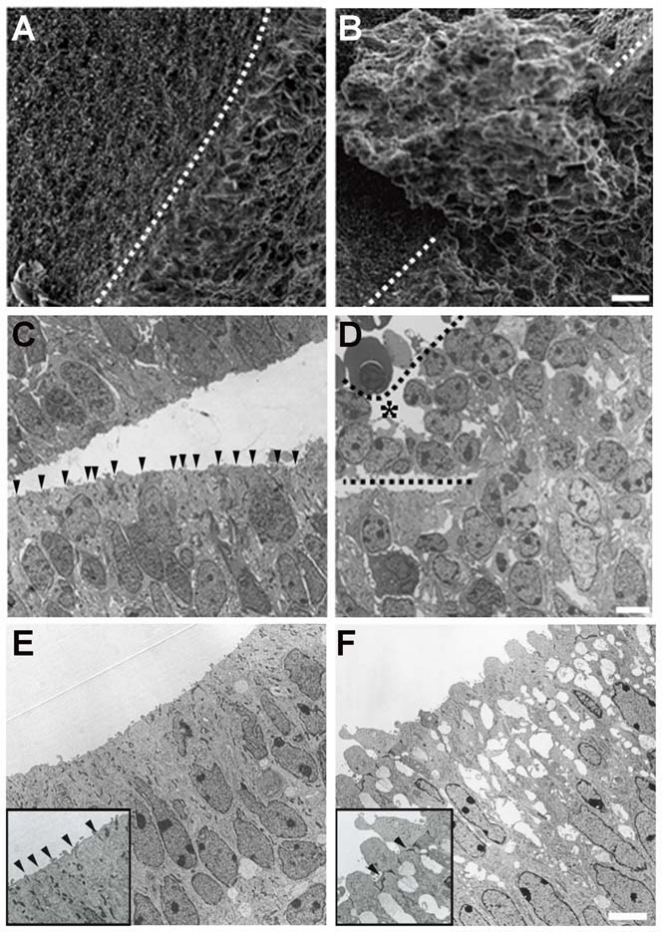
Widespread disruption of neuroepithelium architecture in mDia-DKO mice. (A, B) Scanning electron micrograph of the surface of the lateral ventricle wall from wild-type (A) and mDia-DKO (B) mice at E16. Note that neuroepithelial cells protruded into the lateral ventricle in mDia-DKO mice. Dotted line indicates the cut edge of the ventricle wall. (C–F) Transmission electron micrograph of neuroepithelial cells lining the ventricle wall from wild-type (C, D) and mDia-DKO (E, F) mice at E16. An asterisk indicates a region of periventricular dysplastic mass marked by dotted lines. Arrowheads indicate apical adherens junction. Insets in E and F show higher magnification of the apical surface of neuroepithelium. (A, B) Scale bar, 10 µm. (C, D) Scale bar, 5 µm. (E, F) Scale bar, 5 µm.

We next examined the architecture of the neuroepithelium in mDia-DKO mice in more detail by the use of electron microscopy, because the neuroepithelial cells in areas other than those with the denudation and misalignment appeared normal in mDia-deficient neuroepithelial cells at the level of light microscopy. Scanning electron microscopy (SEM) analysis revealed that, in periventricular dysplastic mass of E16 mDia-DKO embryos, neuroepithelial cells lose their regular alignment with apical-basal polarity and protrude into the ventricular space (Fig. 4A, 4B). In addition, rugged apical surface with membrane protrusions was frequently observed in the neuroepithelium outside periventricular dysplastic mass in mDia-DKO mice (Fig. S4). Observation of ultrathin sections with transmission electron microscopy (TEM) revealed that neuroepithelial cells align to make the smooth apical surface with the existence of high-electron density structure of apical adherens junction between adjacent neuroepithelial cells just beneath the apical surface in the wild-type embryos (Fig. 4C). In contrast, in the periventricular dysplastic mass region of mDia-DKO embryos, the adhesion between the neuroepithelial cells was without the high-electron density structure of apical adherens junction, and the cells were rounded and loosely attached each other to make an ectopic mass (Fig. 4D). In most of the remaining regions of the ventricle wall of mDia-DKO embryos, although the adherens junctions were apparently formed beneath the apical surface, they were occasionally distorted and the apical membrane protruded above the adherens junction, suggesting impaired maintenance of apical adherens junction (Fig. 4E, 4F). Notably, many neuroepithelial cells of mDia-DKO embryos showed low-electron density spaces between cells, suggesting shrinkage of neuroepithelial cells (Fig. 4E, 4F). Such low-electron density space was mostly confined to the apical region of the ventricular zone (Fig. S5). These findings by EM suggest that the mDia deficiency impairs the structure of neuroepithelial cells even in a region that appeared normal at the light microscopic level. The finding that the entire neuroepithelial surface is not denuded despite the above cell phenotype indicates possible compensatory mechanism functioning in mDia-DKO mice. To confirm that the function of mDia in the integrity of the apical actin belt is cell-autonomous, we carried out depletion of mDia by RNAi. RNAi of all mDia isoforms by *in utero* electroporation disrupted the apical actin belt at the region where all mDia isoforms were knocked down (Fig. S6). These data suggest that the mDia function is critical in maintenance of the apical actin belt and adherens junction in neuroepithelial cells.

### Abnormal proliferation and differentiation of mDia-deficient neuroepithelial cells

**Figure 5:**
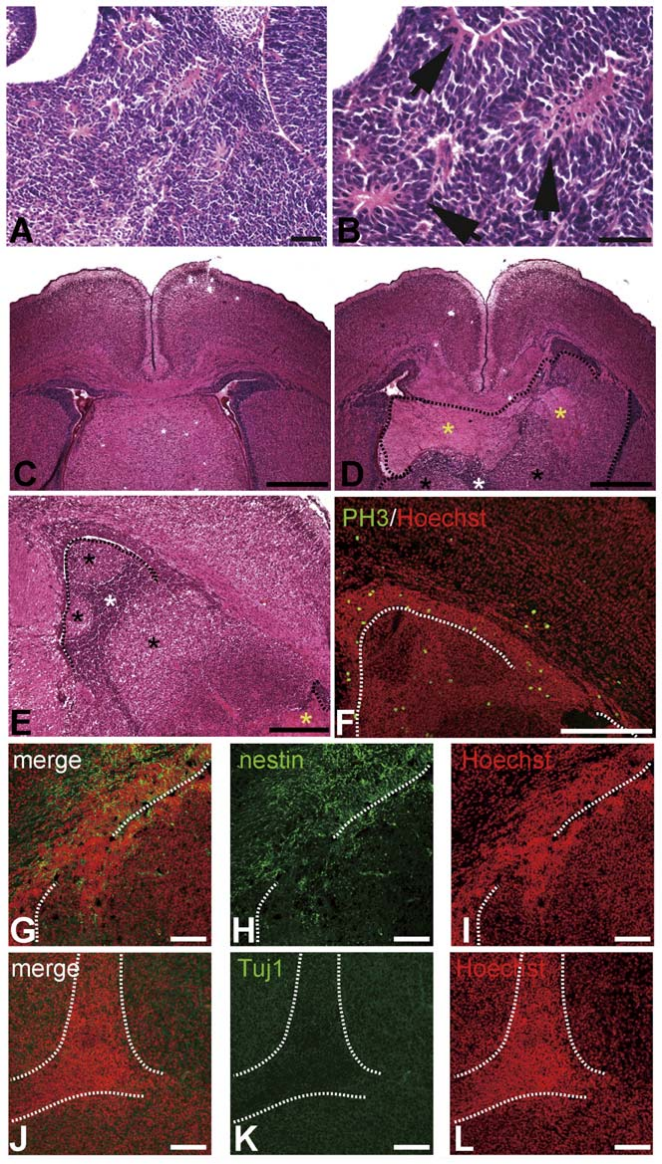
Periventricular dysplastic mass in mDia-DKO mice consists of heterogeneous cell populations of mitotic neural progenitors and non-mitotic neurons. (A, B) Neuro-rosettes in periventricular dysplastic mass of mDia-DKO mice. Neuro-rosette cells (arrows in B) are arranged around a central space filled with fibrillar extensions of the cells (B). A large number of cells with condensed chromatin presumably undergoing mitosis surrounded the fibrillar extensions of neuro-rosettes. (A, B) Scale bars, 50 µm. (C, D) H&E staining of coronal brain sections from control mDia3^null^ (C) and mDia-DKO (D) mice at P0. A region of periventricular dysplastic mass is outlined by dotted lines. Note large periventricular dysplastic mass that completely occupies the lateral ventricle in one hemisphere and disrupts the continuity of the ventricular zone/subventricular zone of the lateral ventricle. (C, D) Scale bars, 500 µm. (E) Higher magnification of H&E staining of a coronal brain section of mDia-DKO mouse at P0. periventricular dysplastic mass, a boundary of which is outlined, is composed of heterogeneous cells of different H&E staining patterns. White, black and yellow asteriks (D and E) indicate the cell populations with dense, light nuclear staining and strong eosin staining, respectively. (E) Scale bar, 250 µm. (F) Phospho-histone H3 (PH3) immunoreactivity (green) and Hoechst nuclear staining (red). Mitotic cells are localized in the periventricular dysplastic mass cell population with dense nuclear staining in addition to cells in ventricular zone of lateral wall and basal progenitors. (F) Scale bar, 100 µm. (G–I) Nestin immunoreactivity (green) and Hoechst nuclear staining (red) in periventricular dysplastic mass. Dotted lines show the boundary between periventricular dysplastic mass and the ventricular zone. (J–L) Tuj1 immunoreactivity (green) and Hoechst nuclear staining (red) in periventricular dysplastic mass. Dotted lines encircle the dense nuclear staining cell mass. Cells with dense nuclear staining in the periventricular dysplastic mass were nestin positive, while cells with lighter nuclear staining were Tuj1 positive. (G–L) Scale bars, 100 µm.

We found that in the periventricular dysplastic mass of mDia-DKO embryos, there are numerous neuro-rosettes (Fig. 5A). Higher magnification of neuro-rosettes revealed large numbers of cells undergo mitosis with condensed chromatin (Fig. 5B). Immunostaining showed that neuro-rosettes in periventricular dysplastic mass contain several mitotic progenitor cells positive for phospho-histone H3 (PH3), a mitotic marker, but few Tuj1-positive differentiated neurons (Fig. S7). As development proceeds, we found that seven out of ten mDia-DKO mice around birth exhibited large periventricular dysplastic mass that almost completely occupied the lateral ventricle in one hemisphere (Fig. 5C, 5D). In some cases, periventricular dysplastic mass develops to occupy Monro's foramen, a narrow canal linking lateral ventricle and third ventricle (Fig. S8). H&E staining revealed that the periventricular dysplastic mass is composed of at least 3 types of cluster of heterogeneous cell populations with one with dense hematoxylin nuclear staining, one with light hematoxylin nuclear staining and one with strong eosin staining (Fig. 5D and 5E). Cells positive for PH3 (Fig. 5F), and for nestin, a neural progenitor marker (Fig. 5G, 5H, 5I), were seen mostly in the cluster of the dense nuclear staining population in the periventricular dysplastic mass, suggesting their neural progenitor character. On the other hand, the cells with light nuclear staining surrounding the above nestin-positive cell mass in periventricular dysplastic mass were negative for nestin and positive for Tuj1, suggesting that they represent differentiated neuronal population (Fig. 5J, 5K, 5L). These results suggest that periventricular dysplastic mass in mDia-DKO mice is an ectopic mass composed of mitotic neural progenitors surrounded by differentiated neuronal cells.

We then examined cell cycle dynamics in periventricular dysplastic mass by EdU labeling and immunostaining for PH3 and Ki67 in E13 brains of control and mDia-DKO mice. Whereas the periventricular dysplastic mass contains ectopic neural progenitors positive for PH3, the proportion of PH3-positive cells per total cells were smaller in the periventricular dysplastic mass than in the neuroepithelial cells of control and mDia-DKO mice (Fig. S9). To examine whether the cell cycle dynamics was altered in periventricular dysplastic mass cells, we analyzed the proportion of the S phase in the entire cell cycle by measuring the proportion of cells labeled by a 60-min pulse of EdU in Ki67-positive proliferating cells. The proportion of the S phase was the same in periventricular dysplastic mass cells and neuroepithelial cells of control and mDia-DKO mice (Fig. S10). On the other hand, the proportion of EdU-labeled cells that became Ki67-negative in 24 hrs after EdU injection was increased in the periventricular dysplastic mass of mDia-DKO mice (Fig. S11), indicating accelerated exit from the cell cycle in periventricular dysplastic mass. Given that the cell cycle exit was not affected in neuroepithelial cells of mDia-DKO mice (Fig. S11), the disruption of apical adherens junctions appears to promote the cell cycle exit, and consequently differentiation in periventricular dysplastic mass. Consistently, as mice develop to adulthood, neurons positive with markers for both mature excitatory neurons (αCaMKII) [18], [19] and inhibitory neurons (GABA) [20], as well as those for all major subtypes of interneuron (parvalbumin, somatostatin and calretinin) [21] were observed in the periventricular dysplastic mass (Fig. S12, Fig. S13).

We next compared the global gene expression profiles between mDia3^null^ control and mDia-DKO E16 forebrain by gene-chip analysis. We found that expression of 159 genes was increased more than 1.5 fold, and expression of 20 genes was decreased less than 0.7-fold (Table S1 and Table S2) in mDia-DKO compared to the control. With exception of Hes1, none of the major signaling molecules of the Hedgehog or Notch pathway showed significant change in the expression level. We then analyzed the mRNA level of several genes involved in these pathways in mDia-DKO mice using quantitative RT-PCR (Fig. S14). The expression level of Gli1, Hes5, Hes1, NeuroD1, Notch1 and Sox2 was not significantly affected, though the expression of Hes1 showed a tendency of slight increase (P<0.06).

### Requirement of balanced Rho activity for maintenance of the apical adherens junction in neuroepithelial cells

**Figure 6:**
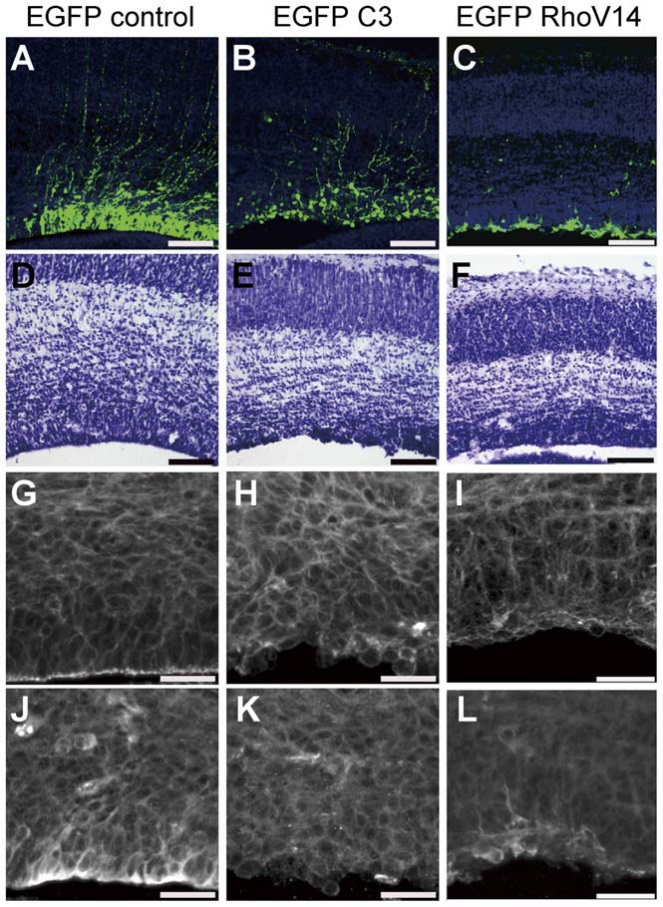
Inactivation or constitutive activation of Rho in neuroepithelial cells disrupts apical F-actin and neuroepithelium integrity. E15 cortices were electroporated with the plasmid expressing EGFP, EGFP-C3 or EGFP-Val14RhoA. At 24 h later, the mice were subjected to histological analyses. (A–C) EGFP (A), EGFP-C3 (B), or EGFP-Val14RhoA (C) was observed with Hoechst (blue) staining in the ventricular zone of the lateral ventricle. In EGFP-C3-positive cells (green), the apical-basal polarity was disrupted. In EGFP-Val14RhoA-positive cells (green), the apical process was lost and the cell body was stacked on the apical surface. The cells also tend to aggregate with each other. (D-F) Nissl staining showed that the apical surface of EGFP-C3-introduced neuroepithelial cells was irregular, with cells protruding into the lateral ventricle. Upon EGFP-Val14RhoA introduction, the ventricular zone was irregular and thinner than those of the control. (G–L) Phalloidin staining (G–I) and immunofluorescent staining for N-cadherin (J–L). The actin filament belt structure and concentration of N-cadherin signals at the apical surface of ventricular zone was lost in both EGFP-C3 (H, K) and EGFP-Val14RhoA-introduced (I, L) cortex. (A–F) Scale bars, 100 µm. (G–L) Scale bars, 25 µm.

Given that mDia1 and mDia3 are Rho effectors, we next determined the role of Rho in neuroepithelial integrity and formation of periventricular dysplastic mass. We introduced botullinum C3 exoenzyme, a Rho inhibitor [22], in neuroepithelial cells by *in utero* electroporation, and observed the morphology of C3-introduced neuroepithelial cells by EGFP. The C3-introduced neuroepithelial cells exhibited a round shape and were tilted with abnormal process(es) projected to random directions (Fig. 6B) in comparison with normal apical-basal polarity of control EGFP-introduced neuroepithelial cells (Fig. 6A). The disruption of the ventricular zone architecture and periventricular dypalsia was observed by Nissl staining (Fig. 6D, 6E). The apical actin filament belt and the apical localization of N-cadherin were lost in the region to which C3 was introduced (Fig. 6G, 6H, 6J, 6K). Simultaneous knockdown of three Rho isoforms, RhoA, RhoB and RhoC, impaired actin filament belt and caused similar disruption of neuroepithelial apical-basal polarity (Fig. S15). Therefore, Rho activity is required for the maintenance of the apical adherens junctions of neuroepithelial cells.

Interestingly, expression of dominant-active Rho also affected the actin structure and the apical localization of N-cadherin (Fig. 6C, 6F, 6I, 6L), suggesting that balanced Rho activity is required for the apical adherens junction of neuroepithelial cells.

To corroborate the importance of the apical actin filament belt of neuroepithelium, we injected *in utero* actin filament-disrupting drugs, cytochalasin D and latrunculin A, into the right lateral ventricle of E15 embryos. Phalloidin staining after 2 h of injection revealed the disruption of apical actin filament belt in the drug-injected ventricle, and the protrusion of the neuroepithelial cells into the ventricular space (n = 6) (Fig. S16A, S16B, S16C, S16D, S16E, S16F). In addition, both compounds induced blood leakage from the choroid plexus on the injected side. On the other hand, pharmacological suppression of ROCK, another Rho effector, had no apparent effect on the neuroepithelium integrity (Fig. S16G, S16H, S16I, S16J, S16K, S16L). Based on these results, we conclude that the apical actin filament belt structure is maintained by constitutive, yet balanced, Rho activity, which provides a support for cell adhesion at the apical surface of neuroepithelial cells.

## Discussion

In the present study, we have generated mice lacking both mDia1 and mDia3, the two isoforms of mDia expressed in the neuroepithelial cells, and have found formation of aberrant periventricular dysplastic mass associated with the hydrocephalus in these mice. The apical actin belt and adherens junction of neuroepithelial cells in the region of periventricular dysplastic mass were disorganized, and the associated apical-basal polarity was lost. In addition, distortion of apical adherens junction, bulging of apical membrane into ventricular space and shrinkage of neuroepithelial cells were widely observed in the remaining region of the ventricular wall throughout the developing brain. Adherens junction components such as N-cadherin and αE-catenin concentrated on the apical surface were previously reported to be involved in the regulation of neuroepithelium structural integrity and apical-basal polarity, and their deletion was found to cause ectopic mass in ventricular space similar to the periventricular dysplastic mass observed in this study [6], [7]. It is known that the filamentous actin belt is associated with such apical adherens junctions in neuroepithelial cells. However, little is known as to how this actin belt is formed, maintained and linked to them. Presumably, the actin belt is organized by the actions of several classes of molecules involved in regulation of actin filament structure, including actin nucleators, actin cross-linking proteins and actin severing proteins [23]. Notably, mutations of filamin-A, an actin cross-linking protein, cause periventricular heterotopia (PVH) in humans [24]. However, what molecules are responsible for *de novo* actin filament formation in neuroepithelial cells is not known. mDia is one of the two major actin nucleators in mammals and known to promote formation of straight filaments [14]. Previous studies utilizing cultured epithelial cells *in vitro* have suggested the possible involvement of mDia1-dependent actin polymerization in the formation and maintenance of the adherens junctions [25]–[27]. However, the role of mDia in adherens junction regulation in the intact body of mammals has not been shown. The present study has utilized mice deficient in mDia and revealed a critical role of mDia in the maintenance of the adherens junction *in vivo*. This study has further shown that this mDia mechanism functions in the specialized structure of the neuroepithelium, the apical adherens junction and the apical actin belt. Consistently, mDia deficiency induces an ectopic mass into the ventricle but the overall cortical structure in mDia-DKO brain is apparently normal in contrast to the N-cadherin and αE-catenin knockout mice that show a severely disorganized cortex [6], [7]. Thus, loss of mDia impairs specifically the apical surface of the neuroepithelium.

Periventricular dysplastic mass induced by mDia deficiency during development is an ectopic mass composed of mitotic neural progenitors and differentiated neurons, suggesting that neural stem cells proliferate and differentiate aberrantly into the ventricular space. Reflecting that the apical actin belt is an important structure for integrity of neuroepithelial cells at all regions of the developing central nervous system, the periventricular dysplastic mass by mDia deficiency occurs in the wall of not only the lateral ventricle but also the third ventricle, aqueduct, fourth ventricle and central canal of spinal cord. This is quite different from clinically observed human PVH that typically arises from the lateral ventricular wall. As development proceeds, neural stem/progenitor cells in periventricular dysplastic mass are eventually differentiated into various types of neurons including αCaMKII positive mature excitatory neurons in adulthood, which is similar to the findings on PVH observed in humans [28]–[30].

mDia is activated by the binding of the GTP-bound form of Rho and exerts its physiological function through the formation of actin filaments. This study has also shown that perturbation of Rho activity causes loss of the apical actin belt and disruption of apical adherens junction in a manner similar to that induced by the loss of mDia. Notably, both suppression and constitutive activation of Rho activity result in disruption of neuroepithelium integrity, indicating that the balance and not mere Rho activation is indispensable. Based on these findings, we conclude that the apical actin belt and the adherens juction in neuroepithelium are maintained by Rho-mDia signaling pathway. Besides, it should be noted that pharmacological inhibition of ROCK, another important Rho effector, with Y-27632 [31], had no apparent effect on the neuroepithelial integrity. ROCK was previously reported to be involved in the closure of the ring-like epithelium structure such as closure of mouse eyelid and ventral body wall [32], [33]. This difference probably reflects the expanding versus contractile nature of the former and the latter epithelial structures, respectively, and suggests that two major signaling pathways of Rho, Rho-mDia and Rho-ROCK, are utilized differently in each of these processes. Such different *in vivo* roles of mDia and ROCK may be consistent with opposing roles of these two molecules on adherens junctions *in vitro* in cultured cells [25].

Complementary to our above findings, Herzog *et al.* have reported during the preparation of our manuscript that conditional deletion of RhoA in spinal cord neuroepithelial cells caused the loss of adherens junctions and severe abnormality of the organization of cells in the spinal neuroepithelium [34]. Katayama *et al.* have also reported after our submission that conditional deletion of RhoA in the midbrain or the forebrain results in similar disruption of adherens junctions, massive expansion of neural progenitors, and disorganization of the brain [35]. However, it should be noted that there are some differences of phenotype between RhoA conditional knockout mice and mDia-DKO mice. While mDia-DKO mice exhibited the lower percentage of PH3-positive cells and an increase of cell cycle exit in the periventricular dysplastic mass and no significant changes in Notch or Sonic-Hedgehog signaling, the latter line of RhoA conditional knockout mice [35] showed the opposite phenotypes. This is probably because the dysplastic mass generated by the loss of RhoA expands not only into the ventricular space but also towards the cortical layer in a similar manner to knockout mice in N-cadherin or αE-catenin as evidenced by the presence of ectopic PH3-positive cells. These results suggest that RhoA uses multiple effector molecules in addition to mDia1/3 to control the overall neuroepithelial cell adhesion, while mDia is involved specifically in the integrity of apical surface. It is interesting that deficiency of mDia1/3 induces widespread abnormality of the apical surface throughout the ventricular wall but causes neuroepithelial dysplasia only in a limited number and places, suggesting the presence of a compensatory redundant mechanism. One possible candidate is mDia2, which is expressed also in developing brain of mice [Shinohara et al., unpublished observation].

Our findings on periventricular dysplastic mass in the third ventricle and Monro's foramen suggest that these masses in mDia-deficient mice could potentially enlarge to occupy ventricular space, obstruct CSF circulation and induce hydrocephalus. We have also observed neuro-rosettes in periventricular dysplastic mass. The neuro-rosettes formation is often observed in primitive neuroepithelial tumors (PNET) [36] and previously reported in clinical hydrocephalus [37]. Whether insufficiency of Rho-mDia signaling pathway is involved in the pathogenesis of PNET and hydrocephalus in humans is currently unknown.

In conclusion, we have demonstrated a critical role of Rho-mDia signaling in the integrity of apical adherens junction and resultant apical-basal polarity of neuroepithelial cells, which is required for neural stem cell homeostasis. Whether mDia-mediated mechanisms play important roles in other stem cell systems should be investigated in the future.

## Materials and Methods

### Animals

Wild-type C57BL/6NCrSlc mice and NZW rabbits were purchased from Japan SLC (Hamamatsu, Japan). mDia1^−/−^ mice were generated as previously described [17]. The generation of mDia3^null^ (^−/−^ or ^−/Y^) mice will be described elsewhere [Shinohara R, *et al.* submitted]. mDia1^−/−^;mDia3^null^ mice were generated as follows. First, mDia3^+/−^ females were crossed with mDia1^+/−^ males. In the offspring, mDia1^+/−^;mDia3^−/Y^ males were mated with mDia1^+/+^;mDia3^+/−^ females, and resultant males and females of mDia1^+/−^;mDia3^null^ genotype were intercrossed to generate mDia1^−/−^;mDia3^null^ (mDia-DKO) mice. Mutant mice used in this study were backcrossed to C57BL/6N genetic background for more than 10 generations. The midday of the day on which a vaginal plug had been detected was designated as embryonic day 0.5 (E0.5). Mice indicated as control in this study are of wild-type or mDia3^null^ genotype. All animal care and use was in accordance with the *National Institutes of Health Guide for the Care and Use of Laboratory Animals* and was approved by the Institutional Animal Care and Use Committee of Kyoto University Graduate School of Medicine (Approval ID: Med Kyo 11036).

### Plasmids

pCX-EGFP, a plasmid expressing EGFP in mammalian cells under CAG promoter [38], is a gift from Dr. Masaru Okabe (Osaka University). To generate pCX-EGFP-botulinum-C3, the fragment containing the open reading frame of EGFP-botulinum-C3 was obtained from pEGFP-botulinum-C3 [39] by PCR with 5′-GAG AAT TCGCCACCA TGG TGA GCA AGG-3′ as a forward primer and 5′-CAG AAT TCC TAT TAT TTA AAT ATC-3′ as a reverse primer. The resultant fragment was inserted into the EcoRI site of pCX-EGFP. To generate pCX-EGFP-Val14RhoA, the fragment containing the open reading frame of EGFP-Val14RhoA was obtained from pEGFP-Val14RhoA [40] by PCR with 5′-GAG AAT TCG CCA CCA TGG TGA GCA AGG-3′ as a forward primer and 5′-CAG AAT TCC TAT CAC AAG ACA AGG CAA CCA GAT-3′ as a reverse primer and then inserted into the EcoRI site of pCX-EGFP. To construct shRNA-expressing plasmids, oligonucleotides targeting the coding region of mouse mDia1 (5′- GGA CAA GTT TGT TGA GAA G-3′), mouse mDia2 (5′-AAG TCA TTG TCC CAG TTT A -3′), mouse mDia3 (5′-GGG CTT GAT ATT CAG TTG A -3′), mouse RhoA (5′-GAC ATG CTT GCT CAT AGT C-3′), mouse RhoB (5′-GCC TAT GAC TAC CTC GAG T-3′), mouse RhoC (5′-CTA TAT AGC CGA CAT CGA A-3′) and a scramble oligonucleotide (5′-GGT ACG GCA ATT CCA CTT T-3′) were designed using BLOCK-iT siRNA Designer (Invitrogen) and modified for shRNA-expressing vector. All of the shRNA contain the sequences for the hairpin loop structure (5′-TTC AAG AGA-3′ or 5′-TTG ATA TCC G-3′). These oligonucleotides were inserted into the pSilencer 2.0 U6 vector (Ambion) between BamHI and HindIII restriction sites. All the plasmids were prepared using EndoFree Plasmid Maxi Kit (Qiagen), and the sequences were confirmed by DNA sequencing.

### Cell culture and transfection

NIH 3T3 cells were maintained in DMEM (Gibco) supplemented with 10% fetal calf serum at 37°C with the atmosphere containing 10% CO_2_. To transfect plasmids, electroporation with 2 pulses of 1100 V for 20 ms was performed with Neon Transfection System (Invitrogen) according to the manufacturer's protocol.

### Production of antibody to mDia3 and Western blot analysis

A GST fusion protein of mouse mDia3 (amino acids 33-295) was produced in *Escherichia coli* BL21 cells, and purified with the use of glutathione-Sepharose 4B beads (Amersham Biosciences). The GST-fused-mDia3-fragment was used as the antigen and injected into rabbits. Embryonic brains were lysed in the lysis buffer containing 50 mM HEPES buffer (pH 7.4), 150 mM NaCl, 0.1% Nonidet P-40, phosphatase inhibitors (PhosSTOP, Roche Diagnostics) and protease inhibitors (Complete Tablet, Roche Diagnostics). NIH 3T3 cells were lysed in the lysis buffer containing 50 mM Tris-HCl (pH 7.5), 150 mM NaCl, 1% Nonidet P-40, 0.5% sodium deoxycholate, and protease inhibitors (Complete Tablet, Roche Diagnostics). Protein concentrations were determined by BCA method (BCA protein assay kit, Thermo Scientific). After denatured in Laemmli buffer containing β-mercaptoethanol at 96°C for 5 min, lysates were subjected to SDS-PAGE and Western blotting. Mouse anti-mDia1 antibody (610849, BD Bioscience), rabbit anti-mDia2 antibody [41], rabbit anti-mDia3 antibody (this study), goat anti-mDia3 antibody (N-15, Santa Cruz), mouse anti-RhoA antibody (sc-418, Santa Cruz), rabbit anti-RhoB antibody (sc-180, Santa Cruz), rabbit anti-RhoC antibody (3430S, Cell Signaling Technology), mouse anti-N-cadherin (610920, BD Bioscience), mouse anti-β-catenin (610153, BD Bioscience), mouse anti-aPKCλ (610207, BD Bioscience), mouse anti-α-tubulin antibody (T9026, Sigma) and mouse anti-GAPDH antibody (AM4300, Ambion) were used as primary antibodies. Signals were detected with ECL Plus Western Blotting Detection System (GE Healthcare).

### Scanning and transmission electron microscopy

The brains of embryos of E16 were fixed in 30 mM HEPES buffer containing 2% glutaraldehyde and 2% paraformadehyde. For scanning electron microscopy, after dissection for lateral ventricle surface exposure, specimens were washed with 30 mM HEPES buffer, post-fixed with 30 mM HEPES buffer containing 2% Osmium tetroxide and then dehydrated with a graded series of ethanol solutions. Specimens were then critical point dried and osmium sputter coated according to standard procedures before examination with a JSM-6320F scanning electron microscope (JEOL) at 5 kV. For transmission electron microscopy, after fixation, the cortex was coronally sectioned at 300 µm with a Linear slicer Pro 7 vibratome (DSK). The samples were next washed with 30 mM HEPES buffer and postfixed in 2% OsO_4_ in 30 mM HEPES buffer at 4°C for 2 h. Thereafter, the samples were washed with 30 mM HEPES buffer, dehydrated with ethanol, and embedded in Epon812 epoxy resin (TAAB). Ultrathin sections were cut with a diamond knife, stained with uranyl acetate and lead citrate, and then observed at 80 kV accelerating voltage using a JEM-1200EX transmission electron microscope (JEOL). Images were processed using Adobe Illustrator CS5 and Adobe Photoshop CS2.

### Histology and immunohistochemistry

After deep anesthesia with intraperitoneal injection of sodium pentobarbital (50 mg/kg), embryo heads were rapidly removed and fixed in 0.1 M phosphate buffer (PB, pH 7.4) containing 4% paraformaldehyde. For paraffin sections, the heads were embedded in paraffin and cut into sections at 5-µm thickness. For frozen sections, the heads were cryoprotected with 0.1 M PB containing 30% sucrose, frozen in Tissue-Tek OCT compound (Sakura) under liquid nitrogen, and then were cut into sections at 16-µm thickness using cryostat. H&E staining and Nissl staining was performed using a standard protocol.

For immunohistochemistry, cryosections were incubated with the blocking buffer (phosphate buffered saline (Nissui) containing 1% normal donkey serum (Sigma) and 0.3% TritronX-100) for 1 h at room temperature, and then incubated with the blocking buffer containing a primary antibody. The primary antibodies used in this study were rabbit anti-mDia3 (this study), mouse anti-N-cadherin (610920, BD Bioscience), rabbit anti-N-cadherin (915-004, Enzo Life Sciences), mouse anti-β-catenin (610153, BD Bioscience), mouse anti-aPKC-λ (610207, BD Bioscience), mouse anti-nestin (MAB353, Millipore), mouse anti-β-III-tubulin (Tuj-1) (MAB1195, R&D Systems), rabbit anti-phospho-histone H3 (06-570, Upstate), mouse anti-CaMKII (6G9, Roche), rabbit anti-GABA (A2052, Sigma), rabbit anti-parvalbumin (ab11427, abcam), rat anti-somatostatin (MAB354, Milipore), mouse anti-calretinin (MAB1568, Millipore), mouse anti-Ki67 (NCL-Ki67-MM1, Novacastra) antibodies. After the primary antibody was removed, the sections were incubated with the blocking buffer containing Cy2- or Cy3-conjugated secondary antibodies to mouse or rabbit IgG (Jackson Immunoresearch). Hoechst 33258 (Invitrogen) or DAPI (Molecular Probes) was used for nuclear staining. Phalloidin conjugated with Alexa 546 or Oregon Green 488 (Invitrogen) was used for staining filamentous actin. Bright field images were acquired using an upright BX-50 microscope (Olympus) equipped with a color CCD camera (Optronics) or CCD camera (Hamamatsu Photonics). Fluorescent images were acquired using the inverted TCS-SP5 confocal microscope (Leica). For all immunostaining experiments, a minimum of three sections obtained from at least 2 embryos per genotype from different litters were analyzed. For quantification of the data, images were analyzed by using Metamorph software (Molecular Devices). All images were processed using Adobe Illustrator CS5 and Adobe Photoshop CS2. Fluorescent images were shown in pseudo-color.

### EdU-labeling and detection of EdU-incorporating cells

EdU (Invitrogen) was dissolved in PBS. Pregnant mice of E12 or E13 were injected intraperitoneally with EdU (15 mg/kg) for labeling. Mice were sacrificed 1 or 24 h after injection and the brain were fixed and processed for cyrosections as described above. EdU detection was performed using a Click-iT EdU Imaging kit (Invitrogen) according to the manufacturer's protocol with minor modification.

### RNA Extraction, DNA Microarray and qRT-PCR

Forebrains were dissected out from E16 embryos and total RNA was extracted using RNeasy Micro Kit (Qiagen). Comprehensive DNA microarray analysis were performed with 3D-Gene (Toray Industries). Microarrays were scanned with the ScanArrayLite Scanner (Perkin-Elmer). qRT-PCR was performed with Lightcycler SYBR Green I marker kit on a Lightcycler Instrument (Roche). Primers for qRT-PCR were as the following, Gli1: 5′-GGC CAA TCA CAA GTC AAG GT-3′ and 5′-TTC AGG AGG AGG GTA CAA CG-3′; Hes5: 5′-AAG AGC CTG CAC CAG GAC TA-3′ and 5′-CGC TGG AAG TGG TAA AGC A-3′; Hes1: 5′-AGA AGA GGC GAA GGG CAA GAA -3′ and 5′-CAT GGC GTT GAT CTG GGT CAT-3′; NeuroD1: 5′-CCC GAG GCT CCA GGG TTA T-3′ and 5′-CCC GCT CTC GCT GTA TGA TT-3′; Notch1: 5′-CAA ACT GGC CTG GGT GGG GAC AT-3′ and 5′-AAA AGG CCA GAA AGA GCT GCC CTG AG-3′; Sox2: 5′-GGC AGC TAC AGC ATG ATG CAG GAG C-3′ and 5′-CTG GTC ATG GAG TTG TAC TGC AGG-3′; GAPDH: 5′-TGA ACG GGA AGC TCA CTG G -3′ and 5′-TCC ACC ACC CTG TTG CTG TA-3′. The intensity relative to GAPDH was calculated, and the fold change relative to the intensity in control embryos is presented.

### *In utero* electroporation

*In utero* electroporation was performed as described previously [42]. Briefly, pregnant mice carrying E15 embryos were deeply anesthetized with intraperitoneal injection of sodium pentobarbital (50 mg/kg). An incision of 1 cm was made on the abdominal wall to access the uterus. Plasmid DNA colored by Fast Green (0.1%) in 0.5 µl was injected by pressure into the right lateral ventricle of an embryo through a glass pipette connected to the Pneumatic PicoPumps (World Precision Instruments). Electroporation (5 pulses of 33 V, 50 ms) was then performed using CUY21 Electroporator (BEX, Tokyo, Japan). After injection, the uterus was carefully returned to the abdominal cavity, and the abdominal incision was sutured. The concentrations of the plasmids are as follows: 1.0 µg/µl (pCX-EGFP, pCX-EGFP-C3 exoenzyme, and pCX-EGFP-Val14RhoA), 0.5 µg/µl (plasmids expressing shRNA for RhoA, RhoB and RhoC), and 1.5 µg/µl (plasmids expressing the scramble shRNA). shRNA-expressing plasmids were co-transfected with pCX-EGFP (0.5 µg/µl) to visualize transfected cells.

### *In utero* intraventricular injection

To intraventricularly inject drugs, 0.5 µl of Y-27632 (100 µM), cytochalasin D (0.5 µg/µl), latrunculin A (0.5 µg/µl) or the vehicle (0.9% NaCl) colored by Fast Green (0.1%) was injected with the same procedure as *in utero* electroporation, except that the electroporation was omitted.

### Statistical analyses

The data are shown as means ± SEM. Statistical significance was determined using a two-tailed Student's t test. Significance was set at *p<0.05, **p<0.01, and ***p<0.001. The analyses were performed by Prism 5.0 software (GraphPad).

## Supporting Information

Figure S1 **Widespread disruption of neuroepithelium integrity in mDia-DKO mice.** (A, B) H&E-stained coronal brain sections at the posterior third ventricle level of control (A) and mDia-DKO (B) adult mice. Note that large periventricular dysplastic mass partially occupied the third ventricle (white dotted line) and the ventricle dilation is observed in mDia-DKO mouse. (C, D) H&E-stained coronal brain sections at the anterior lateral ventricle (LV) level of control (C) and mDia-DKO (D) adult mice. Black dotted line marks the boundary of lateral ventricle. Note that the lateral ventricle on the left side is completely occupied by large periventricular dysplastic mass (dotted line) in mDia-DKO mouse. The boundary of periventricular dysplastic mass was marked by white dotted line. (A–D) Scale bars, 250 µm.
(PDF)

Figure S2 **Periventricular dysplastic mass in the third ventricle, aqueduct and forth ventricle of mDia-DKO mice.** (A, B) H&E-stained coronal sections of the third ventricle wall from control (A) and mDia-DKO (B) mice at E14. In mDia-DKO mice, the third ventricle was dilated and a cell mass containing dense hematoxylin-stained proliferating cells protruded into the ventricle. (C and D) H&E-stained coronal sections of the aqueduct wall from control (C) and mDia-DKO (D) mice at E14. Note that abnormal alignment and protrusion of neuroepithelial cells lining the aqueduct wall was observed in mDia-DKO mice. (E and F) H&E-stained coronal sections of the forth ventricle wall from control (E) and mDia-DKO (F) mice at E14. In mDia-DKO mice, on the left side wall, neuroepithelial aligned abnormally and protruded into ventricular space (arrow). (A–F) Scale bar, 250 µm.
(PDF)

Figure S3 **mDia deficiency do not alter protein expression level of adherens junction components.** Protein expression of N-cadherin, β-catenin, aPKCλ and GAPDH of forebrain lysates from wild-type and mDia-DKO mice at E16. GAPDH was used as an internal control.
(PDF)

Figure S4 **A rugged apical surface of neuroepithelium architecture in mDia-DKO mice.** Scanning electron micrograph of the surface of the lateral ventricle wall from wild-type (A) and mDia-DKO (B) mice at E16 in regions outside periventricular dysplastic mass. Arrows indicate protrusions at the apical surface of neuroepithelial cells in mDia-DKO mice. (A, B) Scale bar, 5 µm.
(PDF)

Figure S5 **Low-electron-density spaces in the apical region of mDia-DKO neuroepithelium.** Transmission electron micrograph of the ventricle wall from wild-type (A) and mDia-DKO (B) mice at E16. Note that abnormal low-electron-density space was localized around the apical surface of the ventricular wall. (A, B) Scale bar, 10 µm.
(PDF)

Figure S6 **mDia depletion by RNAi disrupts apical actin filament and neuroepithelium integrity similarly to mDia-DKO mice.** (A) NIH 3T3 cells were electroporated with plasmids encoding scramble shRNA (lane 1), shRNA's for mDia1 (lane 2), mDia2 (lane 3) or mDia3 (lane 4). Cells were lysed 72 h after electroporation and subjected to Western blotting for mDia1, mDia2, mDia3 and α-tubulin. Endogenous mDia1, mDia2 and mDia3 level were reduced after electroporation with the corresponding shRNA. α-tubulin was used as an internal control. (B) Coronal sections of the lateral ventricle wall at 72 h after electroporation with the plasmid encoding control scramble shRNA or mDia1/2/3 shRNA. EGFP was simultaneously introduced with shRNA to visualize transfected cells. mDia1/2/3 shRNA disrupted apical-basal polarity in neuroepithelial cells. Insets show higher magnification. Scale bar, 100 µm. (C) Phalloidin staining of coronal sections. mDia1/2/3 shRNA significantly reduced the fluorescent signal of the actin filament belt at the apical surface of the ventricular zone. Insets show higher magnification of the apical surface. (B, C) Scale bars, 100 µm.
(PDF)

Figure S7 **Neuro-rosettes contain PH3-positive proliferating cells.** (A–F) Cells in neuro-rosettes from the periventricular dysplastic mass region of mDia-DKO mice at E13 were stained for PH3 (A), Tuj-1 (D) and Hoechst (B and E). C and F represent merged images. White dotted lines (A–F) show boundaries of neuro-rosettes determined by the cell alignment. Yellow dotted lines (A–C) mark the boundaries between periventricular dysplastic mass on the left side and cortex on the right side. (A–F) Scale bars, 50 µm.
(PDF)

Figure S8 **mDia-DKO mice develop large periventricular dysplastic mass that obstruct Monro's foramen.** (A, B) Coronal H&E-stained brain sections from E16 mDia3^null^ control (A) and mDia-DKO mice (B). Asterisk indicates periventricular dysplastic mass in mDia-DKO brain.
(PDF)

Figure S9 **Decreased proportion of PH3-positive mitotic cells in periventricular dysplastic mass of mDia-DKO mice.** (A–C) Immunofluorescent staining for PH3 (green) and nuclear Hoechst staining (blue) of mDia3^null^ control lateral cortex (A), mDia-DKO lateral cortex outside periventricular dysplastic mass (B) and mDia-DKO periventricular dysplastic mass (C, D). periventricular dysplastic mass is indicated by an asterisk and outline by dotted lines in (C). (A–D) Scale bar, 100 µm. (E) Quantification of proportion of the number of PH3-positive mitotic cells to total number of cells is decreased in periventricular dysplastic mass of mDia-DKO mice. Note that no significant difference is observed in neuroepithelial cells of control mice and those of mDia-DKO mice outside the PVH. Five embryos for control (10 sections) and three embryos (8 sections for lateral cortex and 7 sections for periventricular dysplastic mass) for mDia-DKO were analyzed. (F) Quantification of the proportion of the number of mitotic cells to a non-apical region to the total number of mitotic cells of the lateral cortex was quantified in control and mDia-DKO mice. Five embryos for control (10 sections) and three embryos (8 sections) for mDia-DKO were analyzed. The graphs represent mean ± SEM. There is no significant difference. *** P<0.001, n.s.; not significant.
(PDF)

Figure S10 **No alteration in the proportion of S-phase cells in proliferating cells in periventricular dysplastic mass of mDia-DKO mice.** (A–D) Brains were obtained from E13 embryos from wild-type control and mDia-DKO mice 1 h after injection of EdU to pregnant mice. The lateral cortex of a control mouse (A) and an mDia-DKO mouse (B), and periventricular dysplastic mass of mDia-DKO mice (C, D) were stained for EdU (green) and Ki67 (red). Dotted line in (C) shows the boundary between periventricular dysplastic mass and the above cortex. (A–D) Scale bar, 100 µm. (E) Quantification of proportions of the number of EdU-positive cells to the number of Ki67-positive cells in the lateral cortex of control and mDia-DKO mice and periventricular dysplastic mass of mDia-DKO mice. Two embryos for control (3 sections) and two embryos (4 sections for lateral cortex and 6 sections for periventricular dysplastic mass) for mDia-DKO were analyzed. The graphs represent mean ± SEM. n.s.; not significant.
(PDF)

Figure S11 **Accelerated cell-cycle exit of mDia-DKO progenitors in periventricular dysplastic mass.** (A–D) Brains were obtained from E13 embryos from mDia3^null^ control and mDia-DKO mice 24 h after injection of EdU to pregnant mice. The lateral cortex of a control mouse (A) and an mDia-DKO mouse (B), and periventricular dysplastic mass of mDia-DKO mice (C, D) were stained for EdU (green) and Ki67 (red). (A–D) Scale bar, 100 µm. (E) The proportion of the number of EdU-positive and Ki67-negative cells to the total number of EdU-positive cells was quantified in the lateral cortex of control and mDia-DKO mice and periventricular dysplastic mass of mDia-DKO mice. Two embryos for control (4 sections) and two embryos (4 sections for lateral cortex and 5 sections for periventricular dysplastic mass) for mDia-DKO were analyzed. The graphs represent mean ± SEM. * P<0.05, n.s.; not significant.
(PDF)

Figure S12 **Excitatory and inhibitory neurons in periventricular dysplastic mass of mDia-DKO adult brain.** Immunofluorescent staining for αCaMKII (A, E and I) and GABA (B, F and J) with nuclear staining with DAPI (C, G and K) in hippocampal CA3 regions of a control wild-type adult mouse (A–D), an mDia-DKO adult mouse (E–H), and a portion of periventricular dysplastic mass in the lateral ventricle of an mDia-DKO adult mouse (I–L). D, H and L represent merged images. There is no apparently difference between the distribution of excitatory and inhibitory neurons in hippocampal CA3 region between control and mDia-DKO mice. Please note that periventricular dysplastic mass of mDia-DKO mouse consists of clusters of both excitatory and inhibitory neurons. (A–L) Scale bar, 100 µm.
(PDF)

Figure S13 **Interneuron subtypes in PVH of mDia-DKO adult brain.** Immunofluorescent staining for parvalbumin (A, E and I), somatostatin (B, F and J) and calretinin (C, G and K) in coronal sections of hippocampal CA1 regions from a control wild-type adult mouse (A–D), an mDia-DKO adult mouse (E-H), and a portion of periventricular dysplastic mass in the lateral ventricle of an mDia-DKO adult mouse (I–L). D, H and L represent merged images. There is no apparently difference between the distribution of interneuron subtypes in hippocampal CA1 region between control and mDia-DKO mice. Please note that all interneuron subtypes are found in the periventricular dysplastic mass of mDia-DKO mouse. (A–L) Scale bar, 100 µm.
(PDF)

Figure S14 **Expression of genes involved in Hedgehog or Notch signaling pathway in mDia-DKO forebrain.** qRT-PCR analysis of Gli1, Hes5, Hes1, NeuroD1, Notch1 and Sox2 in control and mDia-DKO forebrain at E16. The graphs represent mean ± SEM. n = 3 for control and n = 4 for mDia-DKO embryos. n.s.; non significant.
(PDF)

Figure S15 **Rho depletion by RNAi disrupts apical actin filament and neuroepithelium integrity similarly to EGFP-C3.** (A) NIH 3T3 cells were electroporated with plasmids encoding scramble shRNA (lane 1), shRNA's for RhoA (lane 2), RhoB (lane 3) or RhoC (lane 4). Cells were lysed 72 h after electroporation and subjected to Western blotting for RhoA, RhoB, RhoC and α-tubulin. Endogenous RhoA, RhoB and RhoC were reduced after electroporated with corresponding shRNA. α-tubulin was used as an internal control. (B) Coronal sections of the lateral ventricle wall at 72 h after *in utero* electroporation with the plasmid expressing control scramble shRNA or RhoA/B/C shRNA. EGFP was simultaneously introduced with shRNA to visualize transfected cells. RhoA/B/C shRNA disrupted the apical-basal polarity in neuroepithelial cells. Insets show higher magnification. Scale bar, 100 µm. (C) Phalloidin staining of coronal sections. RhoA/B/C shRNA significantly reduced the fluorescent signal of the actin filament belt at the apical surface of the ventricular zone. Insets show higher magnification of the apical surface. (B, C) Scale bars, 100 µm.
(PDF)

Figure S16 **Effects of actin-perturbing drugs and Y-27632 on the apical actin filament of lateral ventricle wall.** (A–F) Disruption of apical actin filament belt by intraventricular injection of cytochalasin D or latrunculin A. (A–C) Nissl staining of coronal brain sections of E15 embryos injected with control saline (A), or cytochalasin D (B) or latrunculin A (C). Embryos injected with cytochalasin D or latrunculin A showed disruption of the ventricular zone and swelling of the choroid plexus on the injected side. (A–C) Scale bars, 500 µm (A–C). (D–F) Phalloidin staining of coronal brain sections of control saline (D), cytochalasin D (E) and latrunculin A (F) injected embryos. Apical actin filament belt of the lateral wall was severely disrupted on the injected-side (arrows). (D–F) Scale bars, 250 µm. (G–L) Inhibition of ROCK by intraventricular injection of Y-27632 does not apparently affect cortical architecture. E15 wild-type embryos were intraventricularly injected with 0.5 µl of 100 µM Y-27632, a ROCK inhibitor (J, K, L) or saline as control (G, H, I). After 24 h, embryos were collected and analyzed. Coronal sections were stained with phalloidin (H, K and red in I, L) and Hoechst (G, J and blue in I, L). No obvious difference was observed between Y-27632 treated and control embryos. (G–L) Scale bars, 100 µm.
(PDF)

Table S1 **Summary of microarray analysis on gene expression increased in E16 forebrain of mDia-DKO (n = 4) compared to mDia3^null^ (n = 2).** The probe sets up-regulated more than 1.5-fold are listed.
(PDF)

Table S2 **Summary of microarray analysis of on gene expression decreased in E16 forebrain of mDia-DKO (n = 4) compared to mDia3^null^ (n = 2).** The probe sets down-regulated less than 0.7-fold are listed.
(PDF)
