# Supplementary figures and images for: Deficiency of mDia, an Actin Nucleator, Disrupts Integrity of Neuroepithelium and Causes Periventricular Dysplasia
Source: PLoS One. 2011 Sep 28;6(9):e25465. doi: 10.1371/journal.pone.0025465 (PMC3182227; doi:10.1371/journal.pone.0025465)

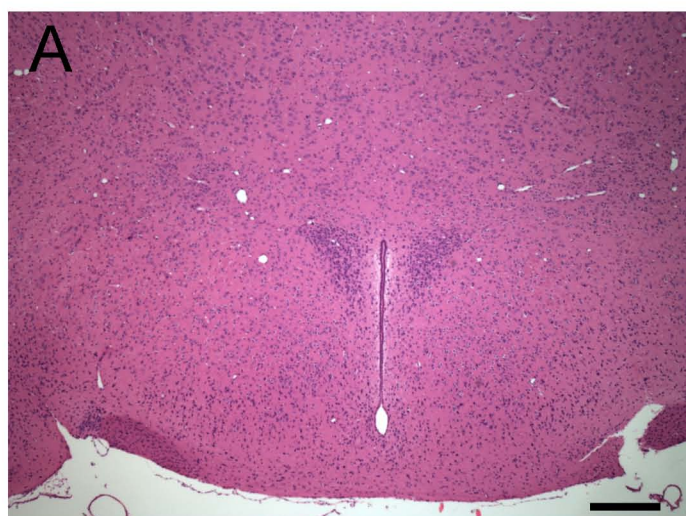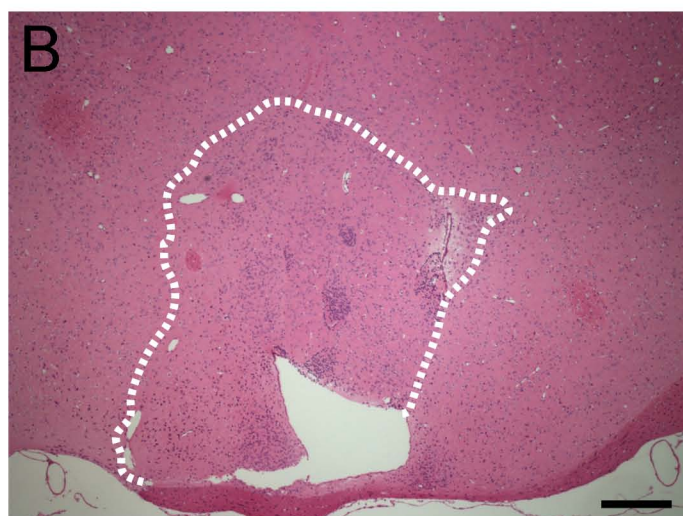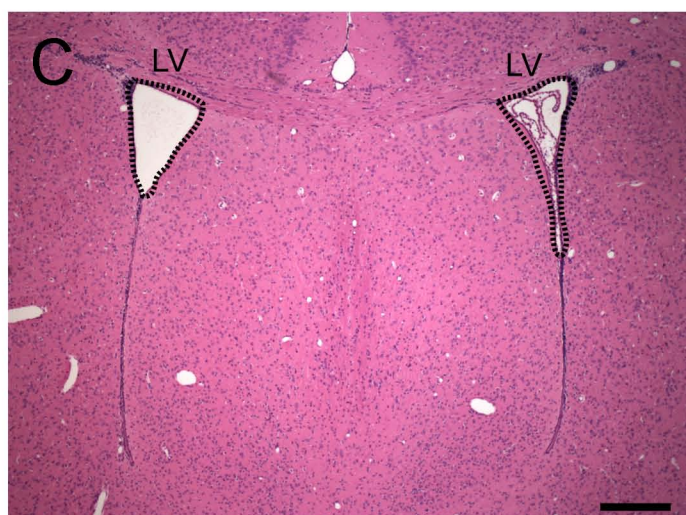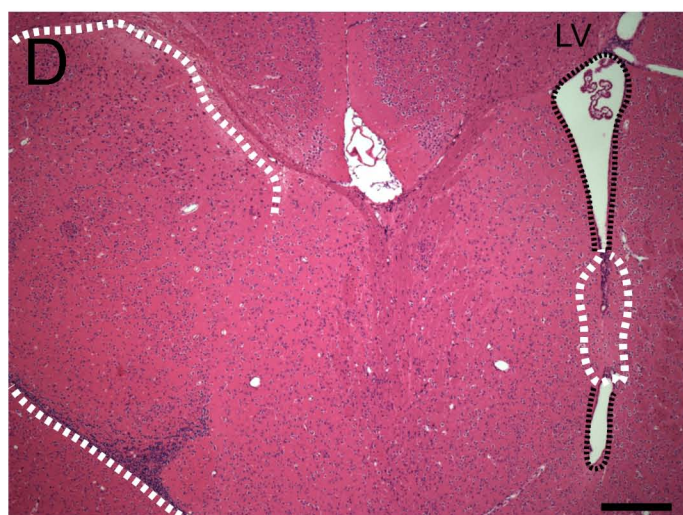

**Figure S1**

Supplement: Figure S1 — Widespread disruption of neuroepithelium integrity in mDia-DKO mice. (A, B) H&E-stained coronal brain sections at the posterior third ventricle level of control (A) and mDia-DKO (B) adult mice. Note that large periventricular dysplastic mass partially occupied the third ventricle (white dotted line) and the ventricle dilation is observed in mDia-DKO mouse. (C, D) H&E-stained coronal brain sections at the anterior lateral ventricle (LV) level of control (C) and mDia-DKO (D) adult mice. Black dotted line marks the boundary of lateral ventricle. Note that the lateral ventricle on the left side is completely occupied by large periventricular dysplastic mass (dotted line) in mDia-DKO mouse. The boundary of periventricular dysplastic mass was marked by white dotted line. (A–D) Scale bars, 250 µm. (PDF) [file pone.0025465.s001.pdf]

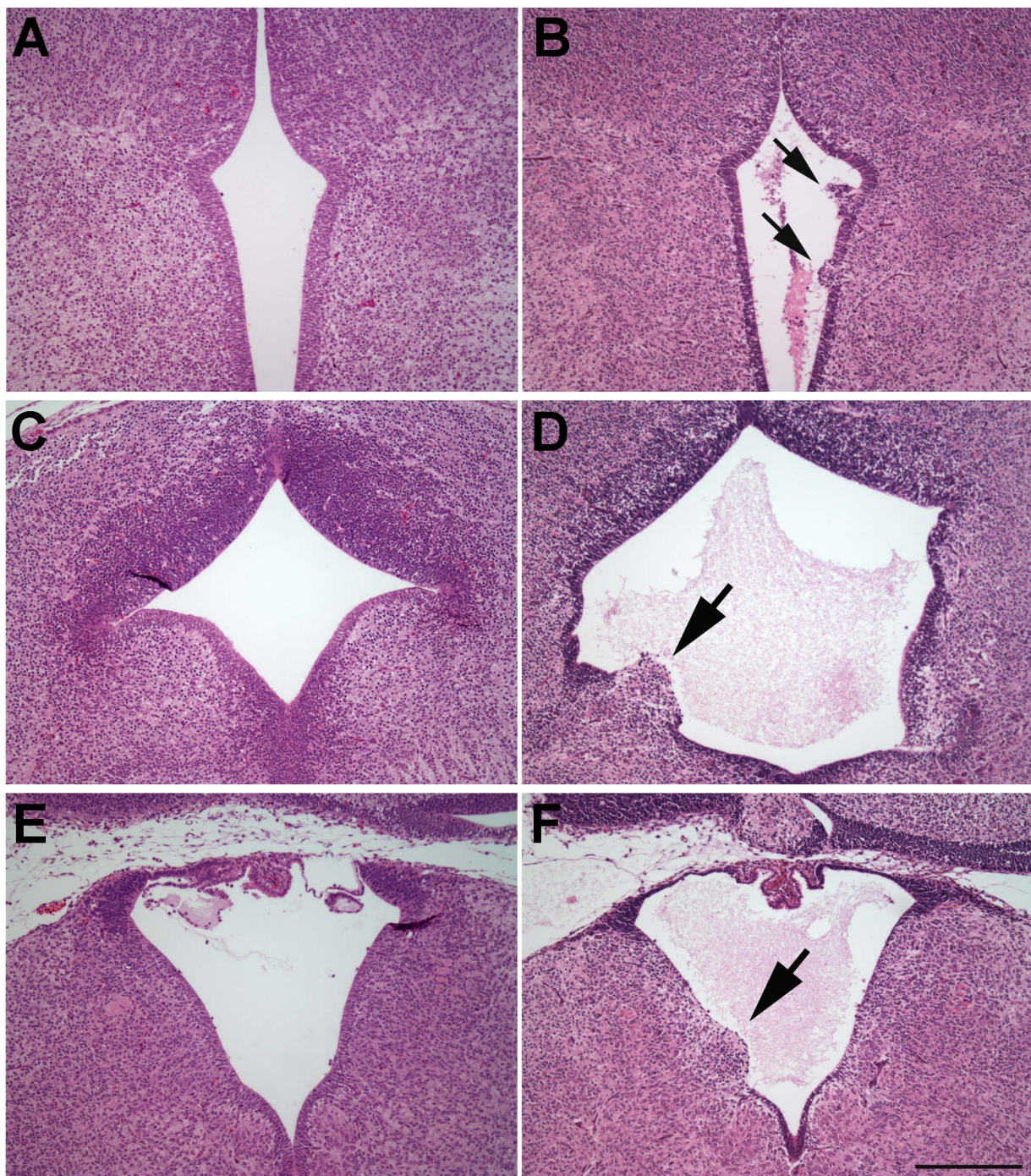

**Figure S2**

Supplement: Figure S2 — Periventricular dysplastic mass in the third ventricle, aqueduct and forth ventricle of mDia-DKO mice. (A, B) H&E-stained coronal sections of the third ventricle wall from control (A) and mDia-DKO (B) mice at E14. In mDia-DKO mice, the third ventricle was dilated and a cell mass containing dense hematoxylin-stained proliferating cells protruded into the ventricle. (C and D) H&E-stained coronal sections of the aqueduct wall from control (C) and mDia-DKO (D) mice at E14. Note that abnormal alignment and protrusion of neuroepithelial cells lining the aqueduct wall was observed in mDia-DKO mice. (E and F) H&E-stained coronal sections of the forth ventricle wall from control (E) and mDia-DKO (F) mice at E14. In mDia-DKO mice, on the left side wall, neuroepithelial aligned abnormally and protruded into ventricular space (arrow). (A–F) Scale bar, 250 µm. (PDF) [file pone.0025465.s002.pdf]

**WT**

**DKO**

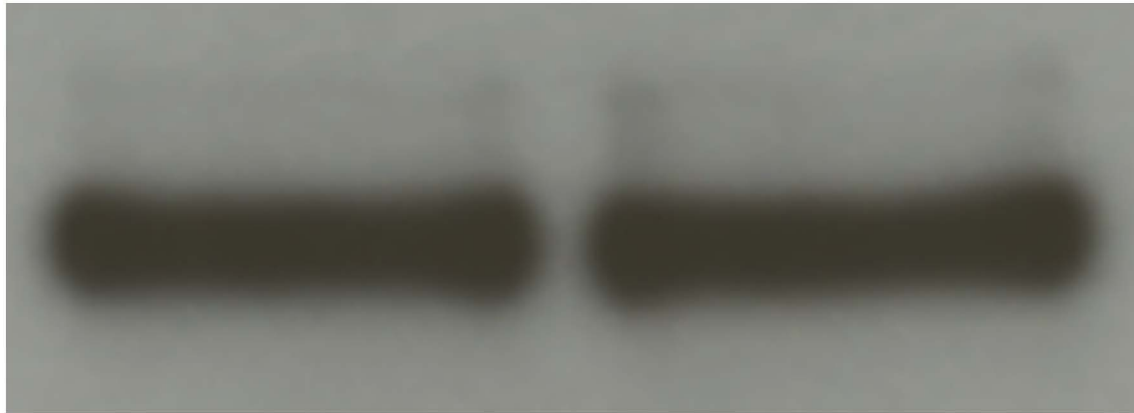

**N-cadherin**

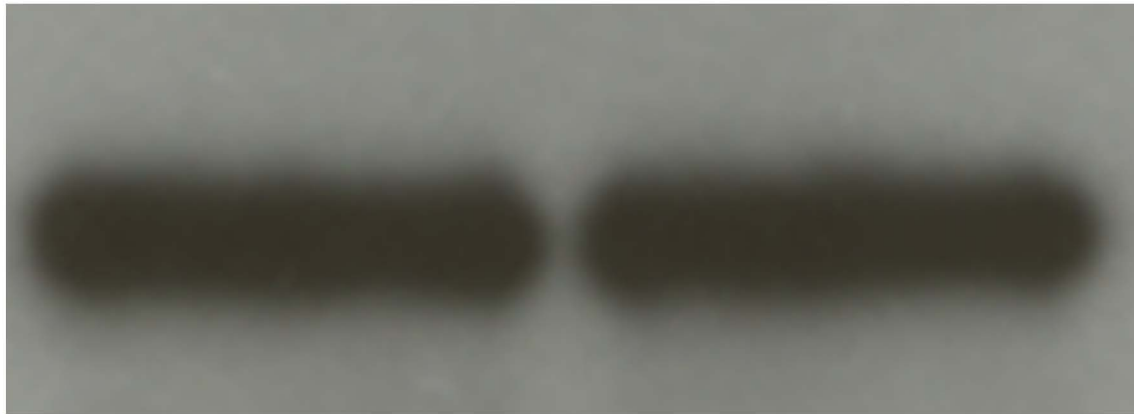

**β-catenin**

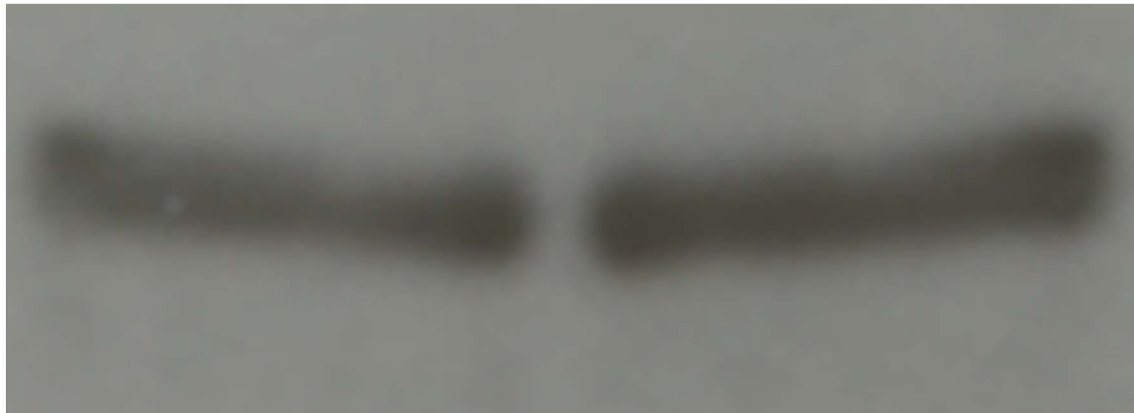

**αPKCλ**

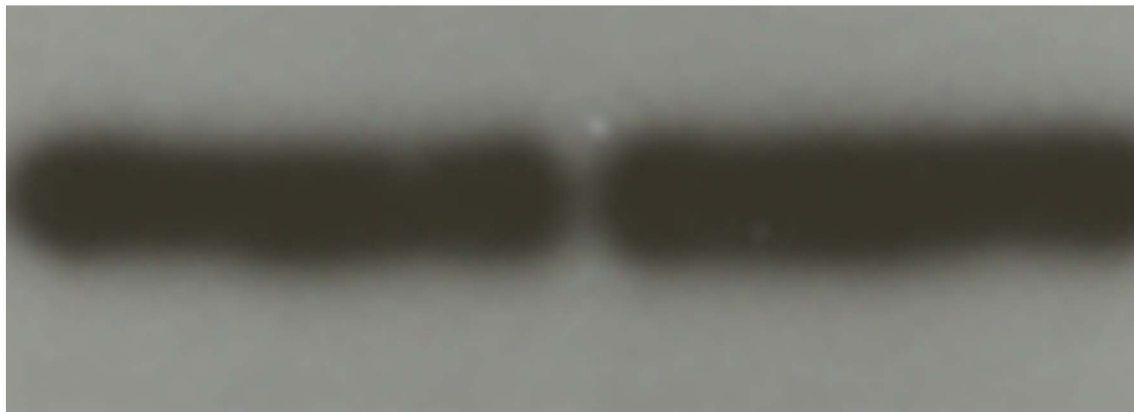

**GAPDH**

**Figure S3**

Supplement: Figure S3 — mDia deficiency do not alter protein expression level of adherens junction components. Protein expression of N-cadherin, β-catenin, aPKCλ and GAPDH of forebrain lysates from wild-type and mDia-DKO mice at E16. GAPDH was used as an internal control. (PDF) [file pone.0025465.s003.pdf]

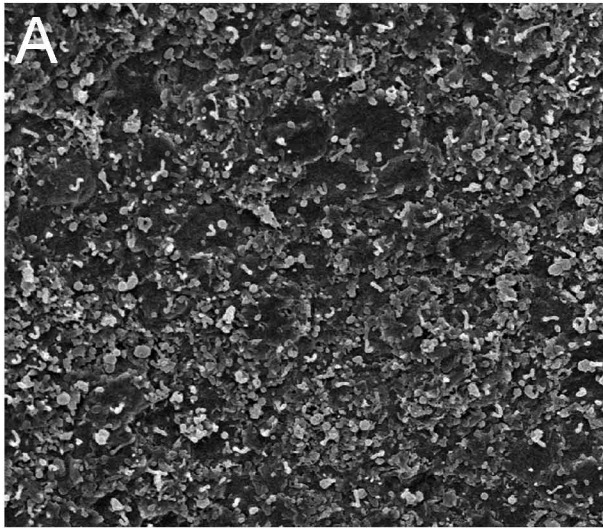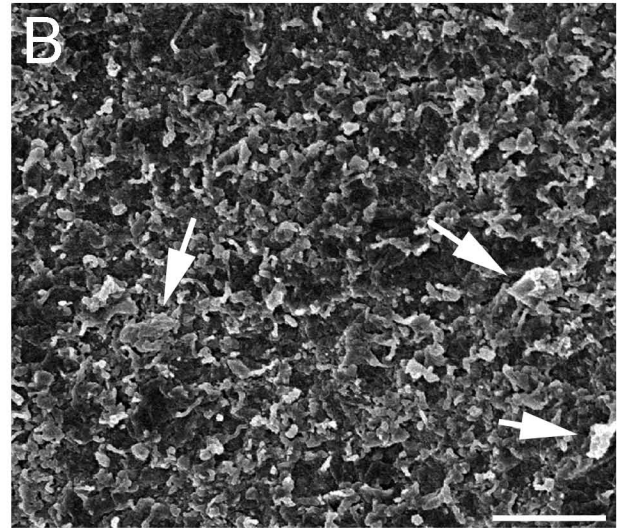

**Figure S4**

Supplement: Figure S4 — A rugged apical surface of neuroepithelium architecture in mDia-DKO mice. Scanning electron micrograph of the surface of the lateral ventricle wall from wild-type (A) and mDia-DKO (B) mice at E16 in regions outside periventricular dysplastic mass. Arrows indicate protrusions at the apical surface of neuroepithelial cells in mDia-DKO mice. (A, B) Scale bar, 5 µm. (PDF) [file pone.0025465.s004.pdf]

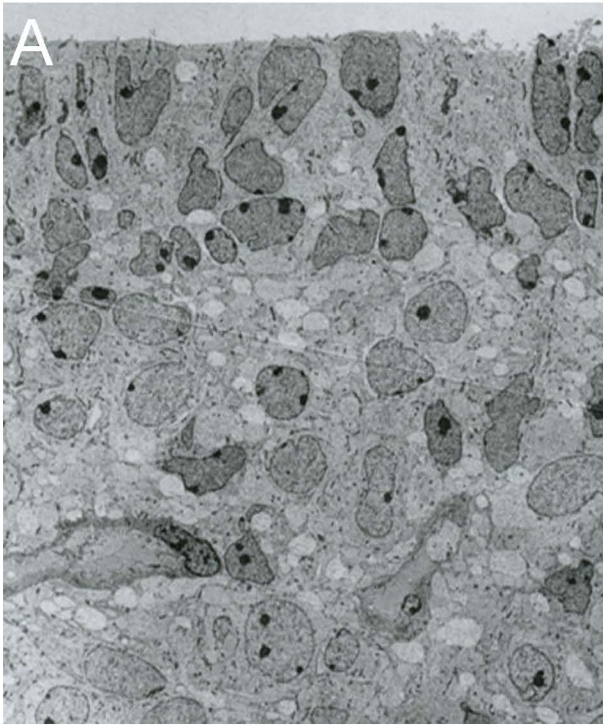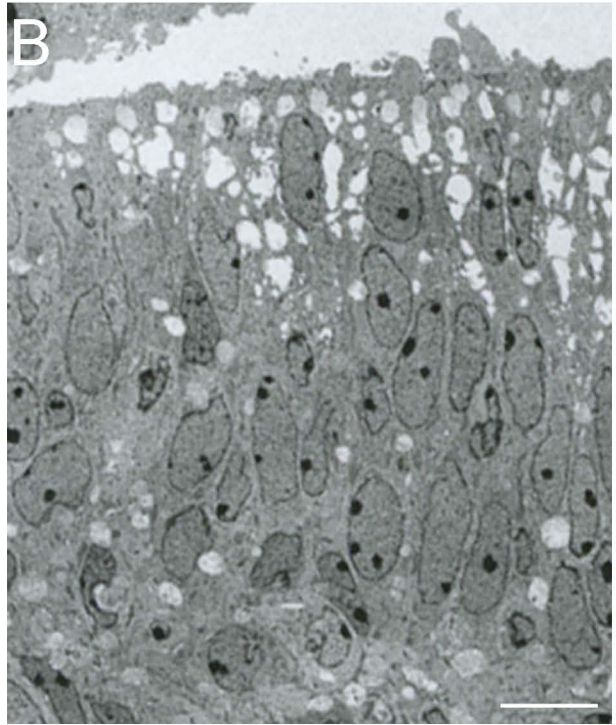

**Figure S5**

Supplement: Figure S5 — Low-electron-density spaces in the apical region of mDia-DKO neuroepithelium. Transmission electron micrograph of the ventricle wall from wild-type (A) and mDia-DKO (B) mice at E16. Note that abnormal low-electron-density space was localized around the apical surface of the ventricular wall. (A, B) Scale bar, 10 µm. (PDF) [file pone.0025465.s005.pdf]

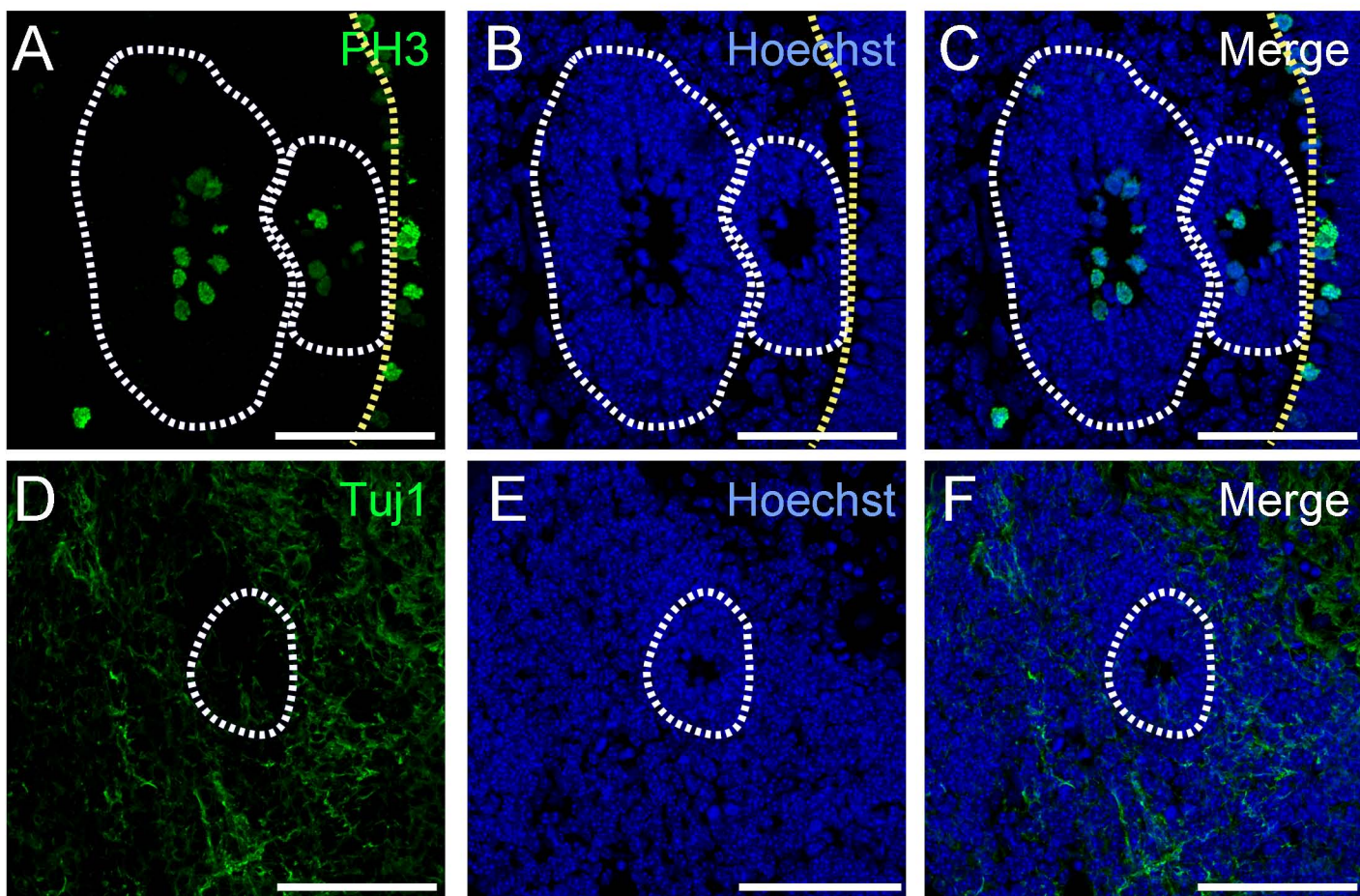

**Figure S7**

Supplement: Figure S7 — Neuro-rosettes contain PH3-positive proliferating cells. (A–F) Cells in neuro-rosettes from the periventricular dysplastic mass region of mDia-DKO mice at E13 were stained for PH3 (A), Tuj-1 (D) and Hoechst (B and E). C and F represent merged images. White dotted lines (A–F) show boundaries of neuro-rosettes determined by the cell alignment. Yellow dotted lines (A–C) mark the boundaries between periventricular dysplastic mass on the left side and cortex on the right side. (A–F) Scale bars, 50 µm. (PDF) [file pone.0025465.s007.pdf]

A

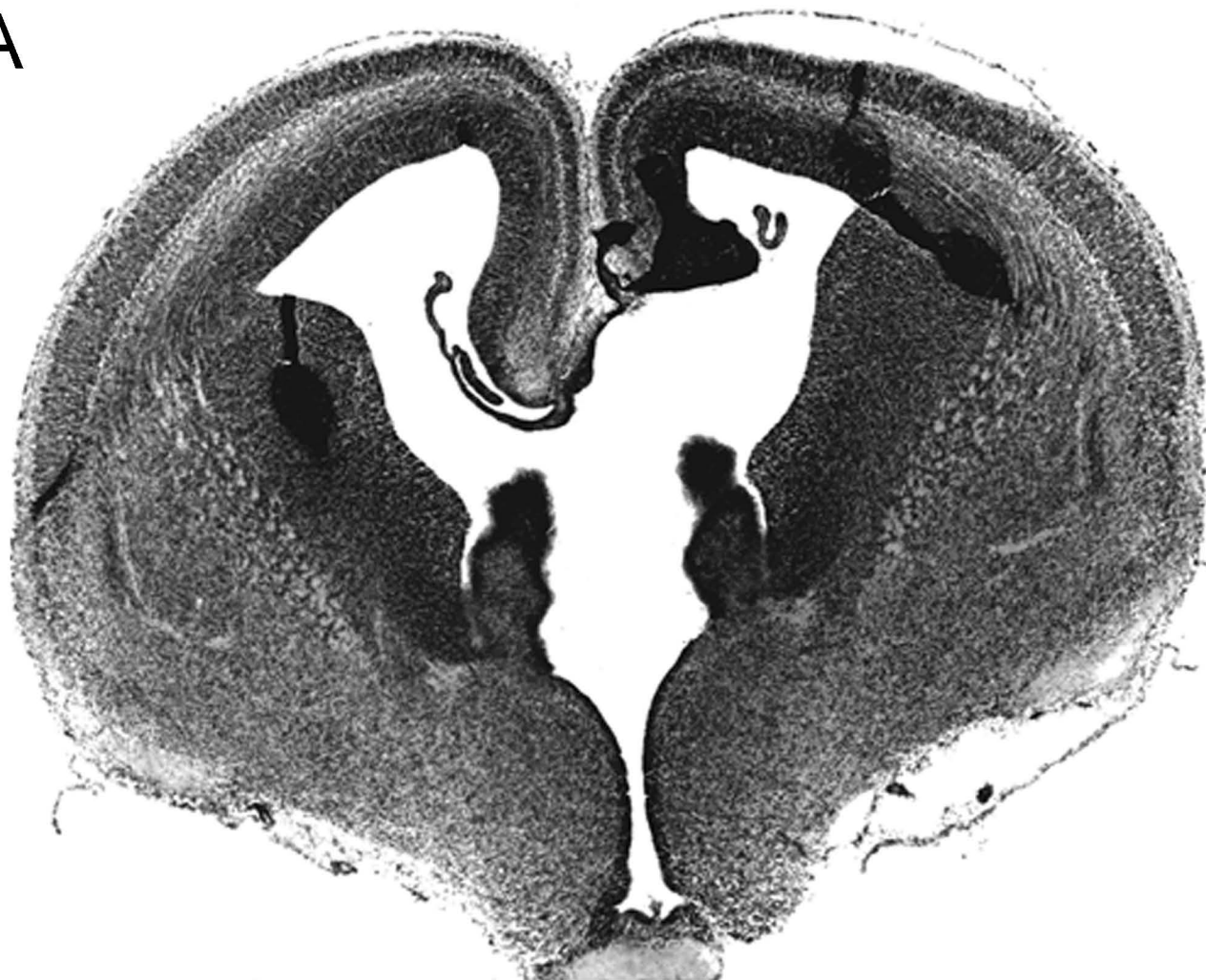

B

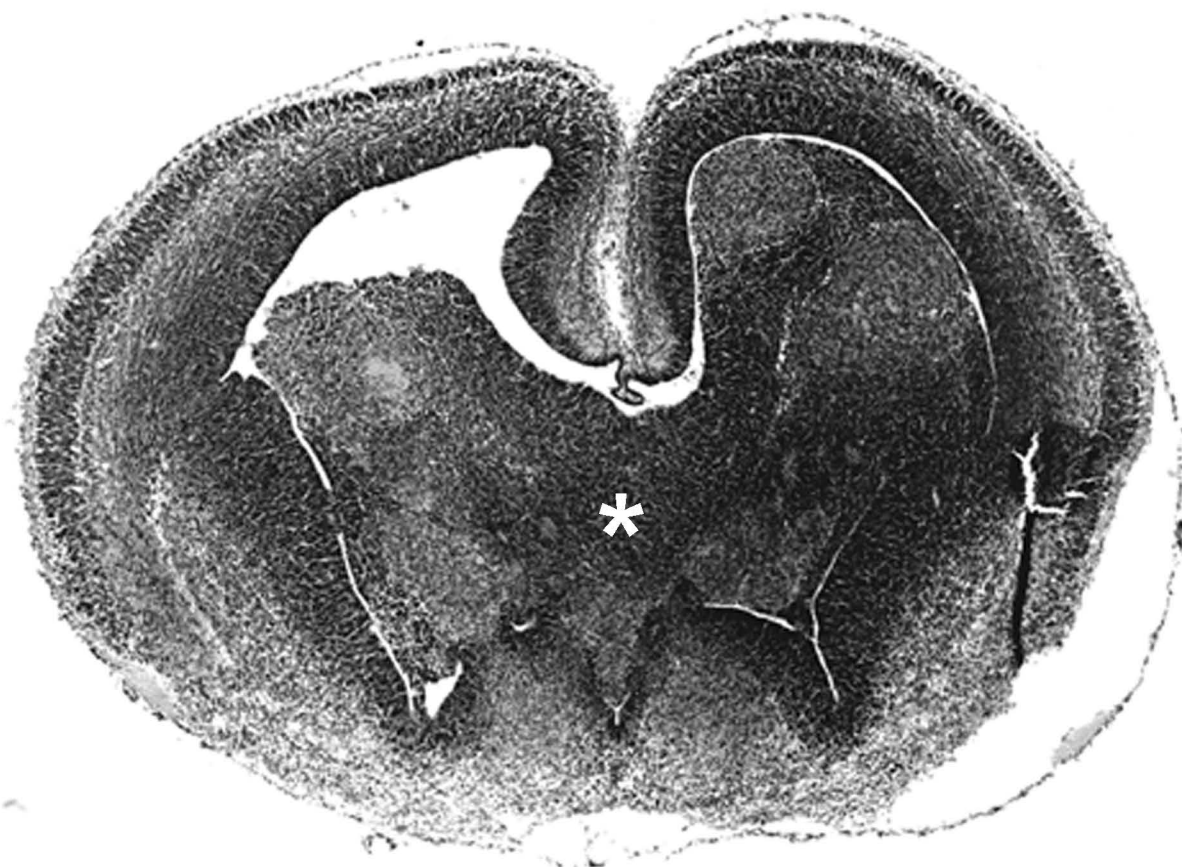

**Figure S8**

Supplement: Figure S8 — mDia-DKO mice develop large periventricular dysplastic mass that obstruct Monro's foramen. (A, B) Coronal H&E-stained brain sections from E16 mDia3null control (A) and mDia-DKO mice (B). Asterisk indicates periventricular dysplastic mass in mDia-DKO brain. (PDF) [file pone.0025465.s008.pdf]

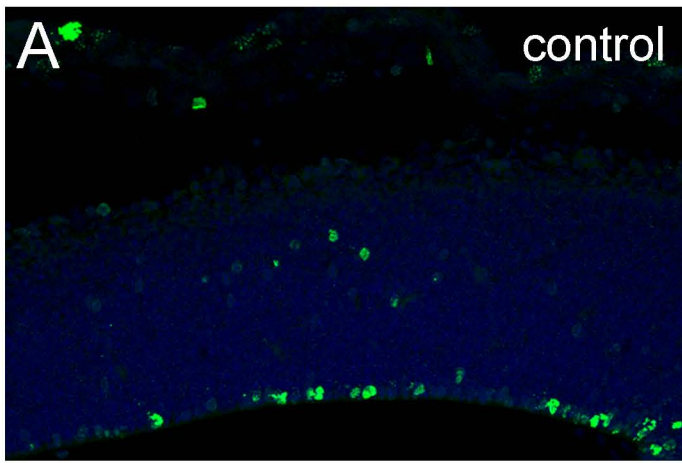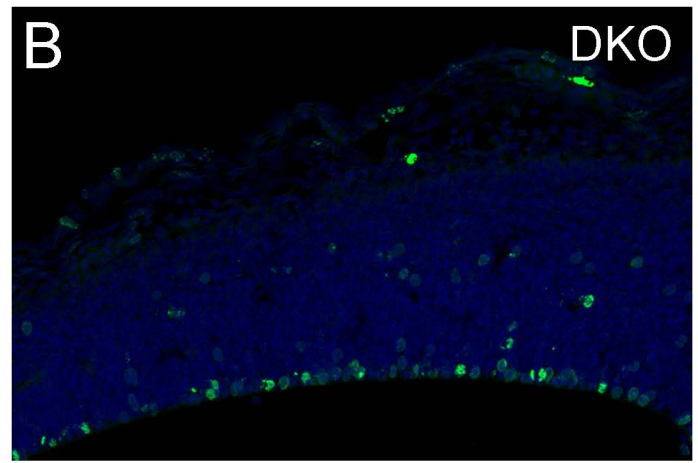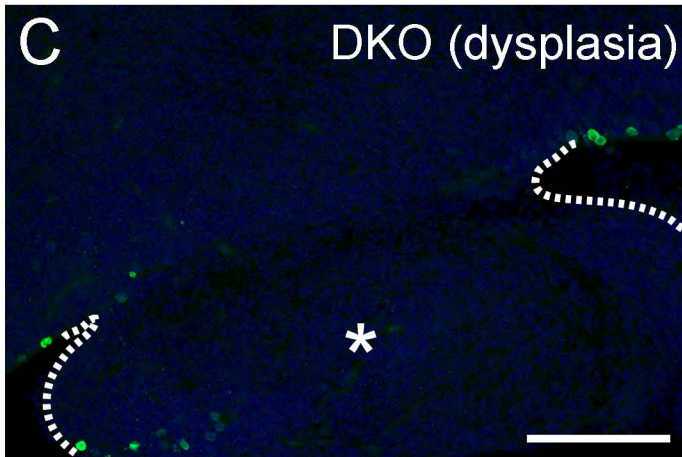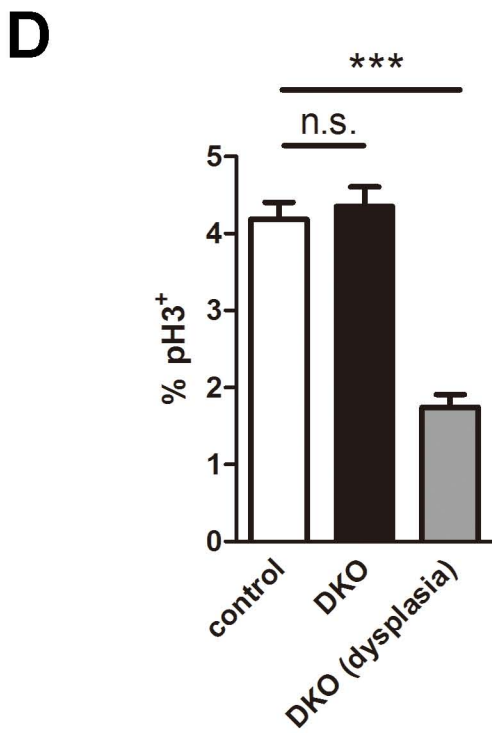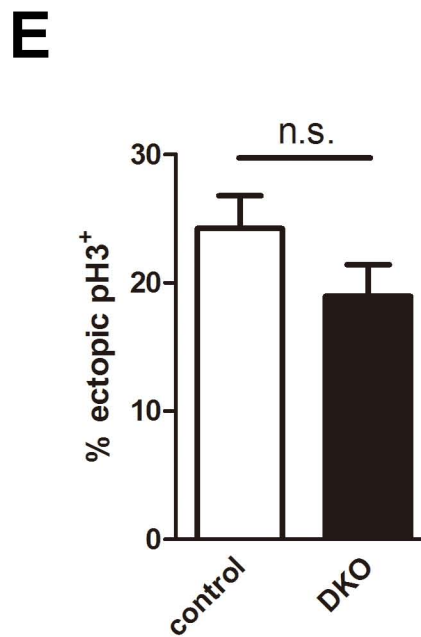

**Figure S9**

Supplement: Figure S9 — Decreased proportion of PH3-positive mitotic cells in periventricular dysplastic mass of mDia-DKO mice. (A–C) Immunofluorescent staining for PH3 (green) and nuclear Hoechst staining (blue) of mDia3null control lateral cortex (A), mDia-DKO lateral cortex outside periventricular dysplastic mass (B) and mDia-DKO periventricular dysplastic mass (C, D). periventricular dysplastic mass is indicated by an asterisk and outline by dotted lines in (C). (A–D) Scale bar, 100 µm. (E) Quantification of proportion of the number of PH3-positive mitotic cells to total number of cells is decreased in periventricular dysplastic mass of mDia-DKO mice. Note that no significant difference is observed in neuroepithelial cells of control mice and those of mDia-DKO mice outside the PVH. Five embryos for control (10 sections) and three embryos (8 sections for lateral cortex and 7 sections for periventricular dysplastic mass) for mDia-DKO were analyzed. (F) Quantification of the proportion of the number of mitotic cells to a non-apical region to the total number of mitotic cells of the lateral cortex was quantified in control and mDia-DKO mice. Five embryos for control (10 sections) and three embryos (8 sections) for mDia-DKO were analyzed. The graphs represent mean ± SEM. There is no significant difference. *** P<0.001, n.s.; not significant. (PDF) [file pone.0025465.s009.pdf]

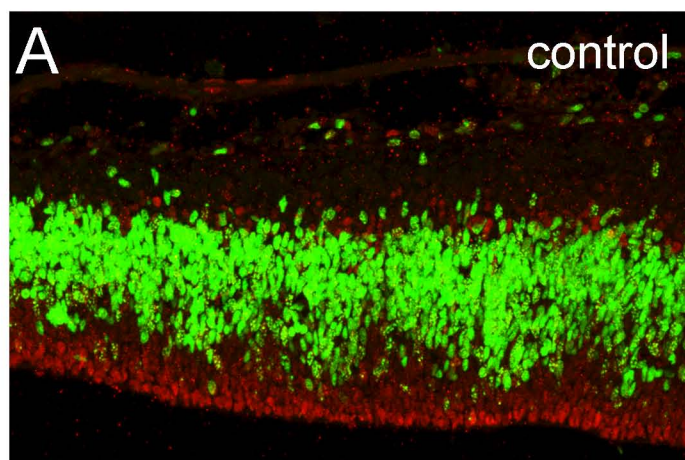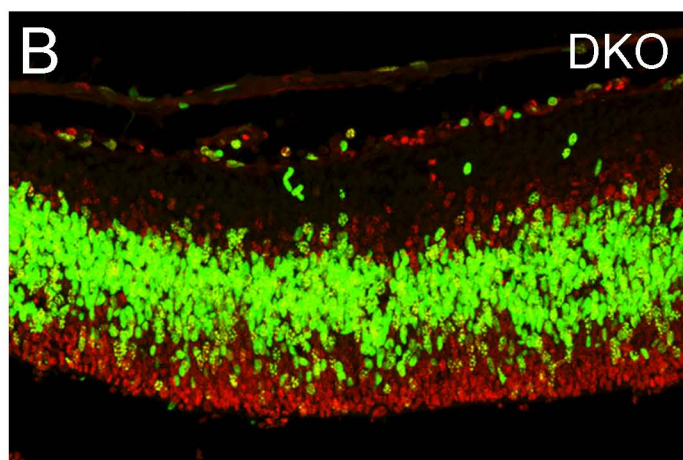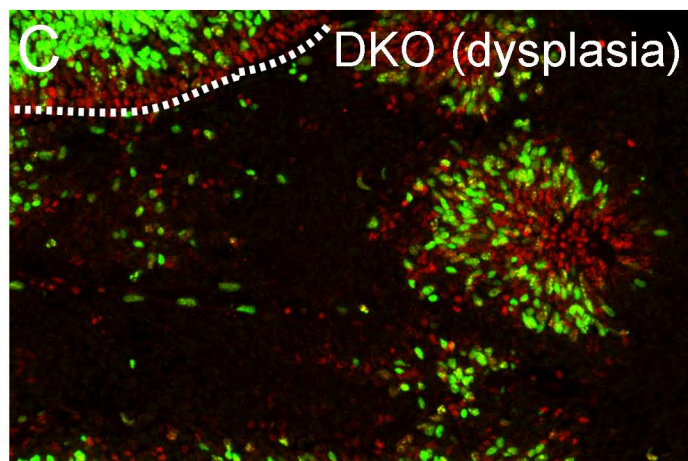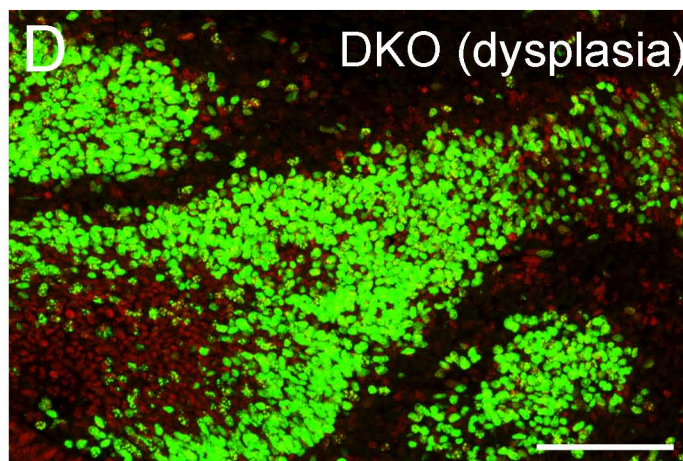

**E**

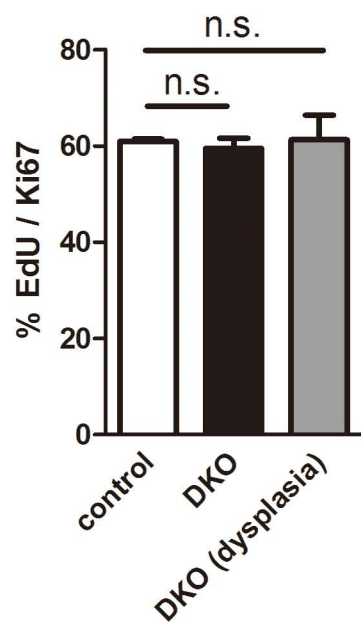

**Figure S10**

Supplement: Figure S10 — No alteration in the proportion of S-phase cells in proliferating cells in periventricular dysplastic mass of mDia-DKO mice. (A–D) Brains were obtained from E13 embryos from wild-type control and mDia-DKO mice 1 h after injection of EdU to pregnant mice. The lateral cortex of a control mouse (A) and an mDia-DKO mouse (B), and periventricular dysplastic mass of mDia-DKO mice (C, D) were stained for EdU (green) and Ki67 (red). Dotted line in (C) shows the boundary between periventricular dysplastic mass and the above cortex. (A–D) Scale bar, 100 µm. (E) Quantification of proportions of the number of EdU-positive cells to the number of Ki67-positive cells in the lateral cortex of control and mDia-DKO mice and periventricular dysplastic mass of mDia-DKO mice. Two embryos for control (3 sections) and two embryos (4 sections for lateral cortex and 6 sections for periventricular dysplastic mass) for mDia-DKO were analyzed. The graphs represent mean ± SEM. n.s.; not significant. (PDF) [file pone.0025465.s010.pdf]

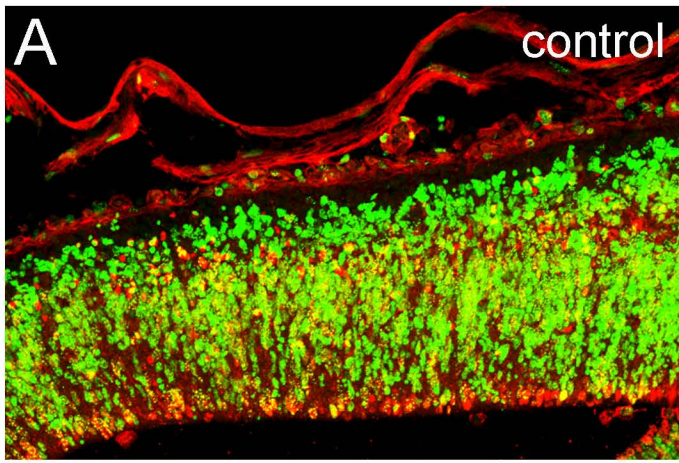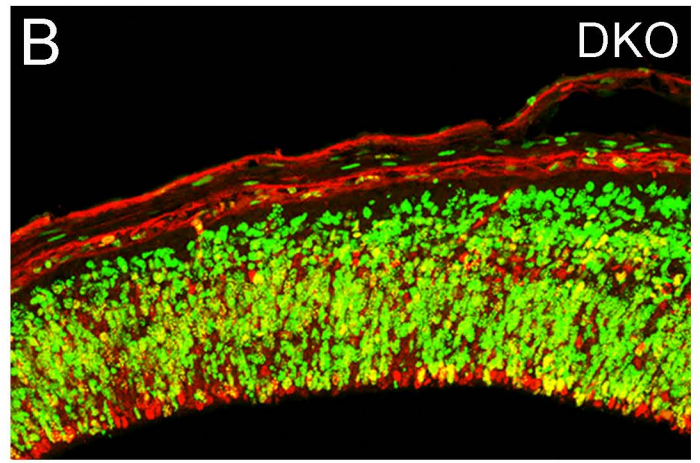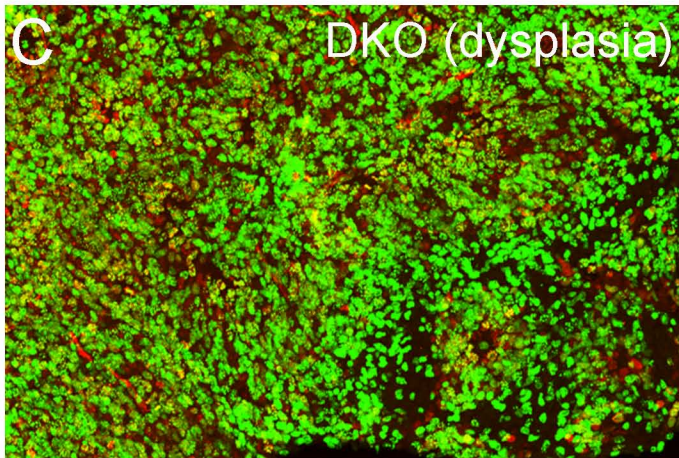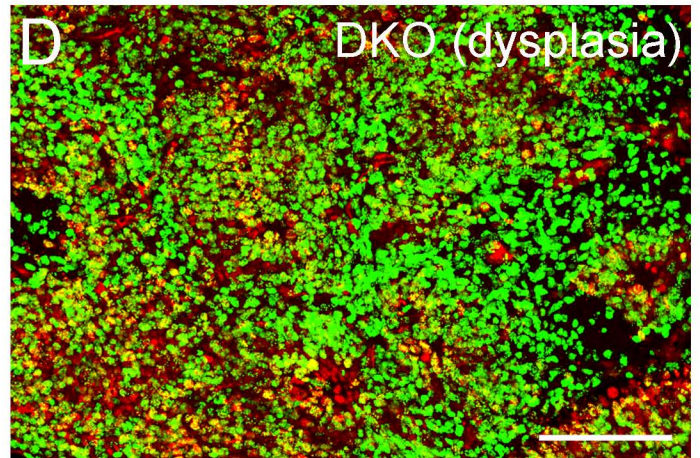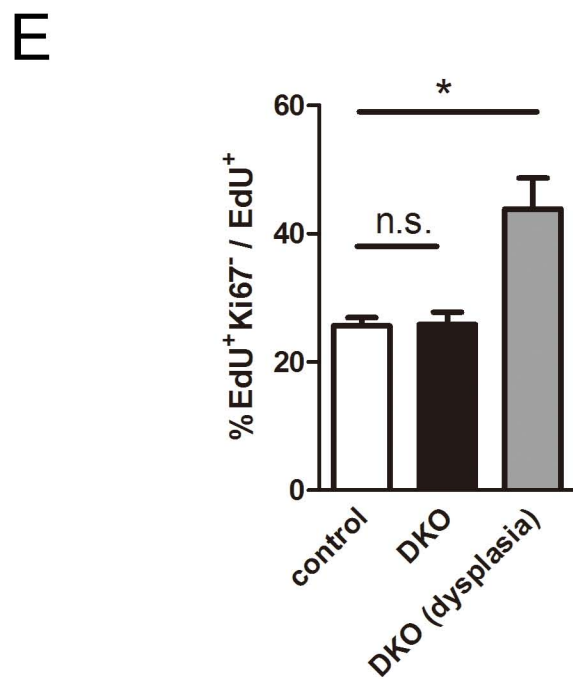

**Figure S11**

Supplement: Figure S11 — Accelerated cell-cycle exit of mDia-DKO progenitors in periventricular dysplastic mass. (A–D) Brains were obtained from E13 embryos from mDia3null control and mDia-DKO mice 24 h after injection of EdU to pregnant mice. The lateral cortex of a control mouse (A) and an mDia-DKO mouse (B), and periventricular dysplastic mass of mDia-DKO mice (C, D) were stained for EdU (green) and Ki67 (red). (A–D) Scale bar, 100 µm. (E) The proportion of the number of EdU-positive and Ki67-negative cells to the total number of EdU-positive cells was quantified in the lateral cortex of control and mDia-DKO mice and periventricular dysplastic mass of mDia-DKO mice. Two embryos for control (4 sections) and two embryos (4 sections for lateral cortex and 5 sections for periventricular dysplastic mass) for mDia-DKO were analyzed. The graphs represent mean ± SEM. * P<0.05, n.s.; not significant. (PDF) [file pone.0025465.s011.pdf]

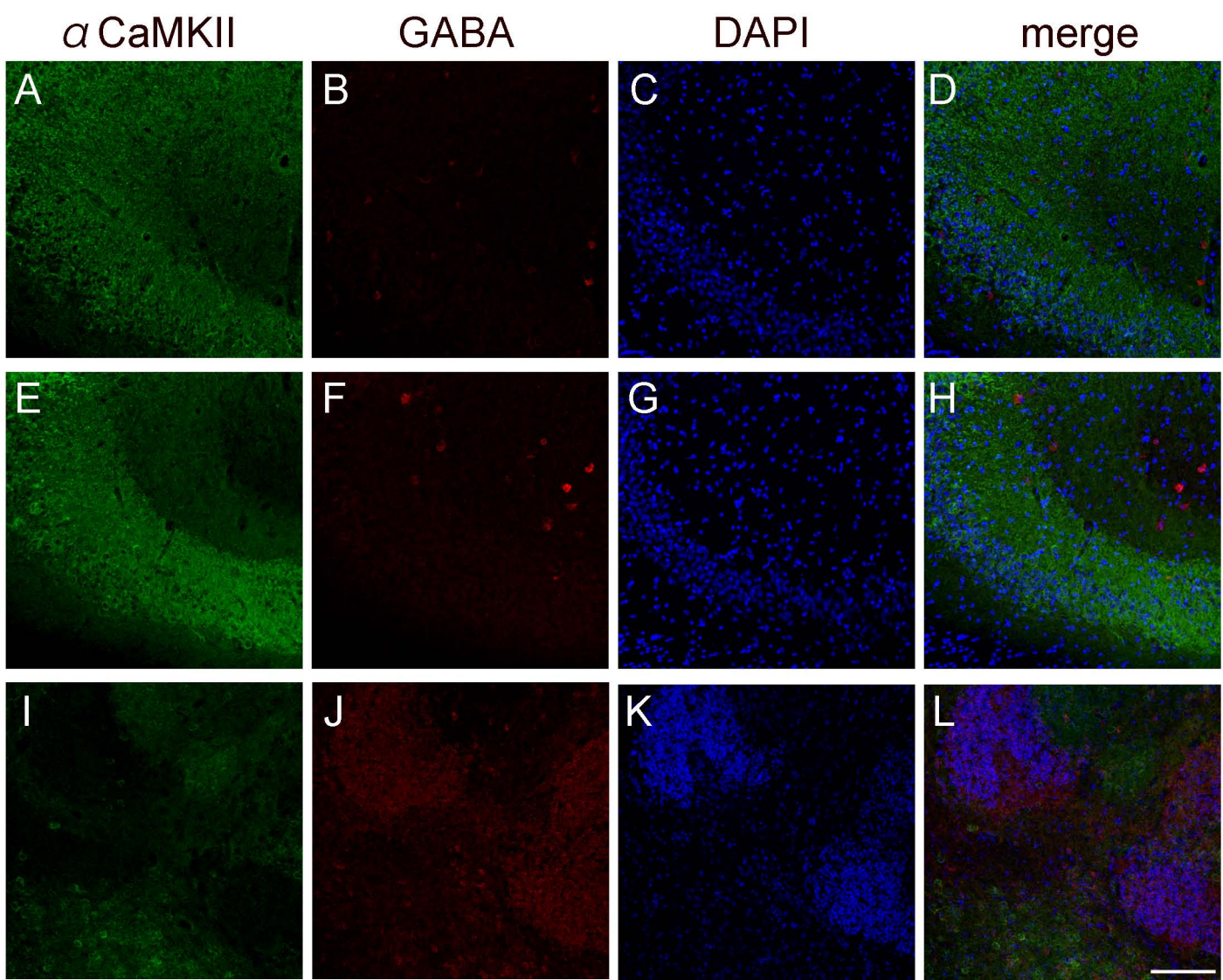

**Figure S12**

Supplement: Figure S12 — Excitatory and inhibitory neurons in periventricular dysplastic mass of mDia-DKO adult brain. Immunofluorescent staining for αCaMKII (A, E and I) and GABA (B, F and J) with nuclear staining with DAPI (C, G and K) in hippocampal CA3 regions of a control wild-type adult mouse (A–D), an mDia-DKO adult mouse (E–H), and a portion of periventricular dysplastic mass in the lateral ventricle of an mDia-DKO adult mouse (I–L). D, H and L represent merged images. There is no apparently difference between the distribution of excitatory and inhibitory neurons in hippocampal CA3 region between control and mDia-DKO mice. Please note that periventricular dysplastic mass of mDia-DKO mouse consists of clusters of both excitatory and inhibitory neurons. (A–L) Scale bar, 100 µm. (PDF) [file pone.0025465.s012.pdf]

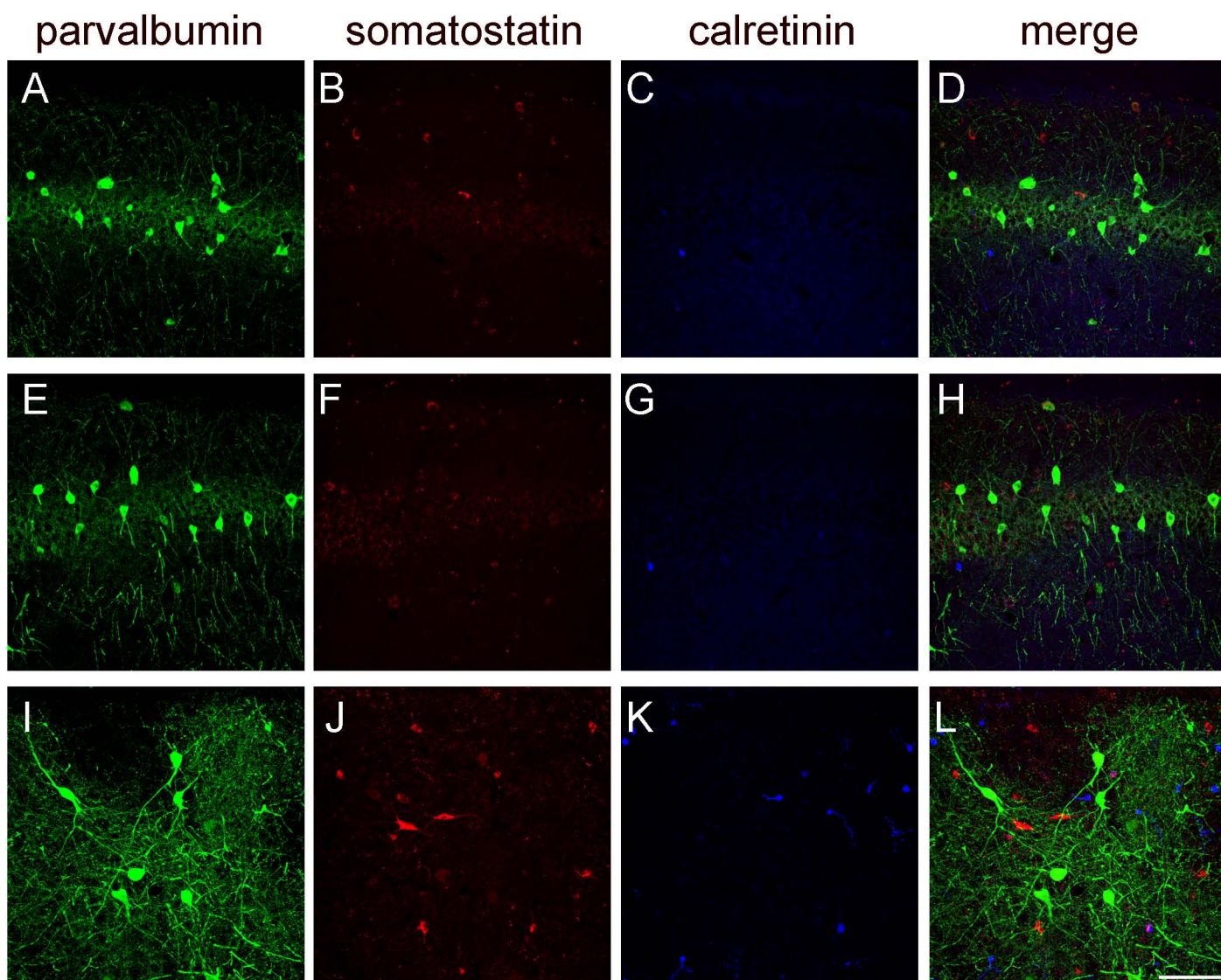

**Figure S13**

Supplement: Figure S13 — Interneuron subtypes in PVH of mDia-DKO adult brain. Immunofluorescent staining for parvalbumin (A, E and I), somatostatin (B, F and J) and calretinin (C, G and K) in coronal sections of hippocampal CA1 regions from a control wild-type adult mouse (A–D), an mDia-DKO adult mouse (E-H), and a portion of periventricular dysplastic mass in the lateral ventricle of an mDia-DKO adult mouse (I–L). D, H and L represent merged images. There is no apparently difference between the distribution of interneuron subtypes in hippocampal CA1 region between control and mDia-DKO mice. Please note that all interneuron subtypes are found in the periventricular dysplastic mass of mDia-DKO mouse. (A–L) Scale bar, 100 µm. (PDF) [file pone.0025465.s013.pdf]

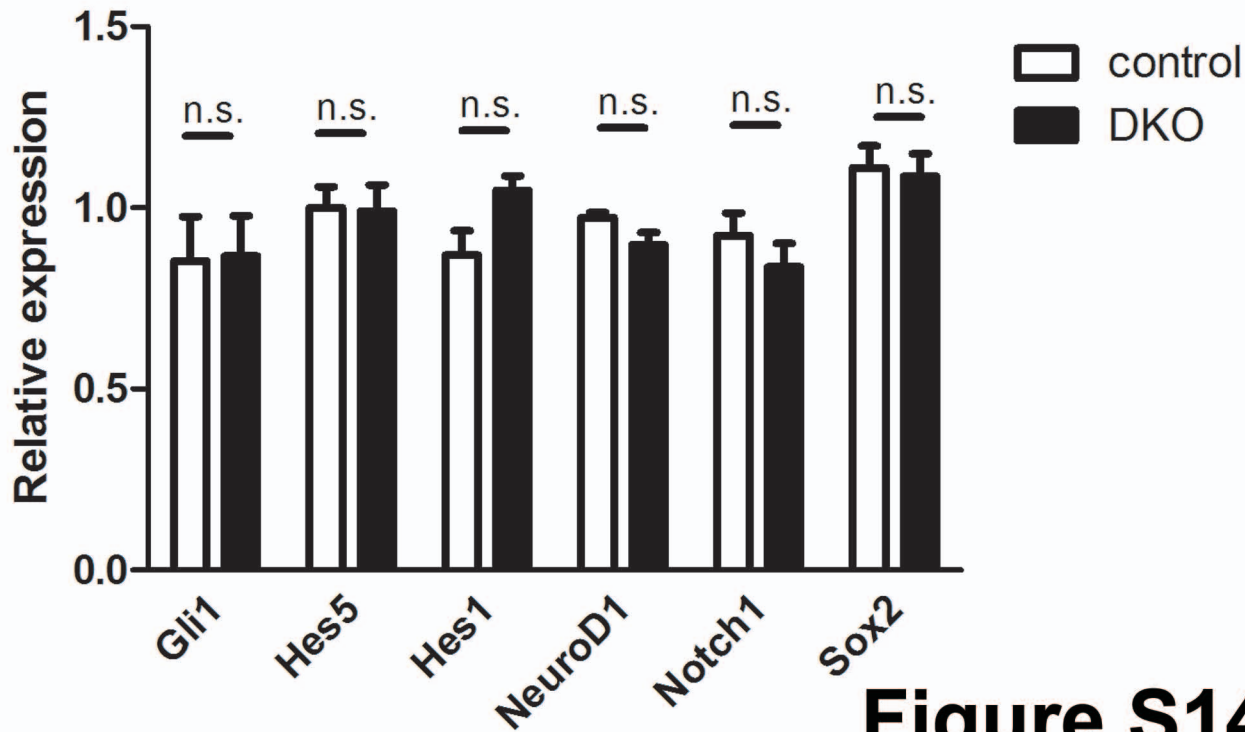

**Figure S14**

Supplement: Figure S14 — Expression of genes involved in Hedgehog or Notch signaling pathway in mDia-DKO forebrain. qRT-PCR analysis of Gli1, Hes5, Hes1, NeuroD1, Notch1 and Sox2 in control and mDia-DKO forebrain at E16. The graphs represent mean ± SEM. n = 3 for control and n = 4 for mDia-DKO embryos. n.s.; non significant. (PDF) [file pone.0025465.s014.pdf]

**A**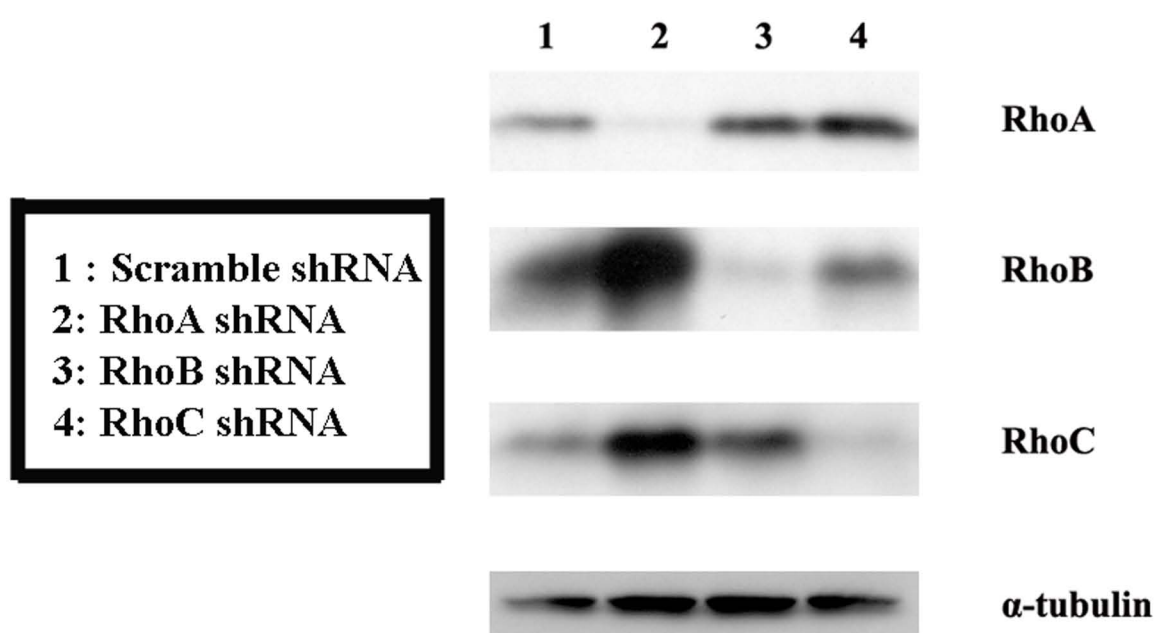**B**

control scramble shRNA

RhoA/B/C shRNA

EGFP

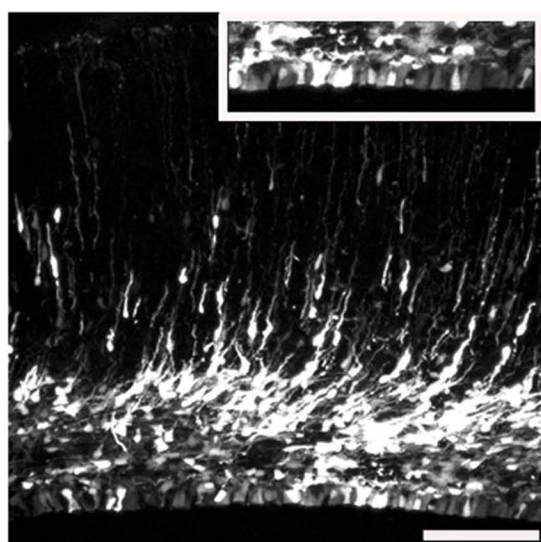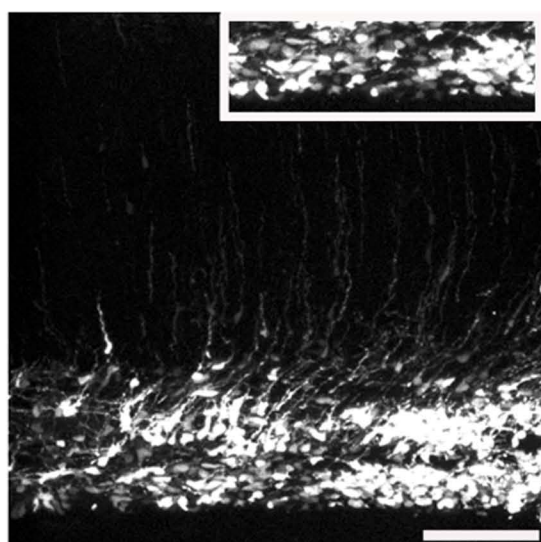**C**

Phalloidin

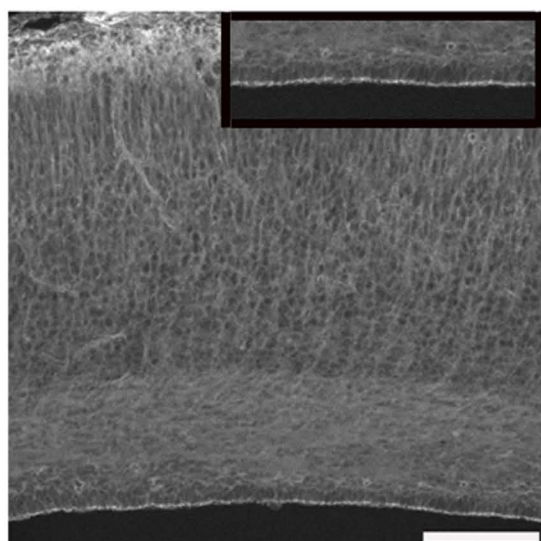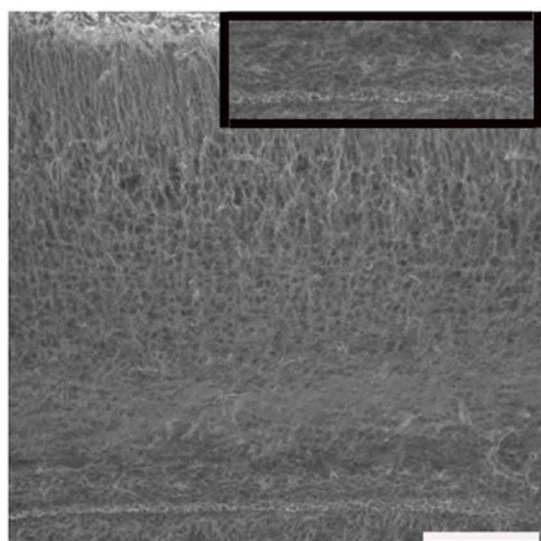**Figure S15**

Supplement: Figure S15 — Rho depletion by RNAi disrupts apical actin filament and neuroepithelium integrity similarly to EGFP-C3. (A) NIH 3T3 cells were electroporated with plasmids encoding scramble shRNA (lane 1), shRNA's for RhoA (lane 2), RhoB (lane 3) or RhoC (lane 4). Cells were lysed 72 h after electroporation and subjected to Western blotting for RhoA, RhoB, RhoC and α-tubulin. Endogenous RhoA, RhoB and RhoC were reduced after electroporated with corresponding shRNA. α-tubulin was used as an internal control. (B) Coronal sections of the lateral ventricle wall at 72 h after in utero electroporation with the plasmid expressing control scramble shRNA or RhoA/B/C shRNA. EGFP was simultaneously introduced with shRNA to visualize transfected cells. RhoA/B/C shRNA disrupted the apical-basal polarity in neuroepithelial cells. Insets show higher magnification. Scale bar, 100 µm. (C) Phalloidin staining of coronal sections. RhoA/B/C shRNA significantly reduced the fluorescent signal of the actin filament belt at the apical surface of the ventricular zone. Insets show higher magnification of the apical surface. (B, C) Scale bars, 100 µm. (PDF) [file pone.0025465.s015.pdf]

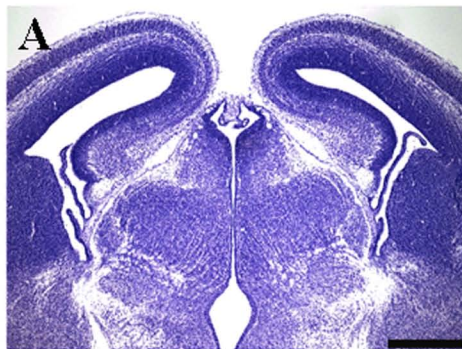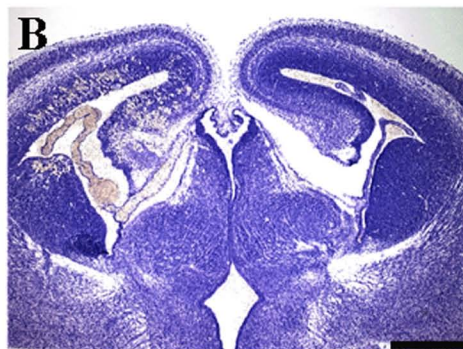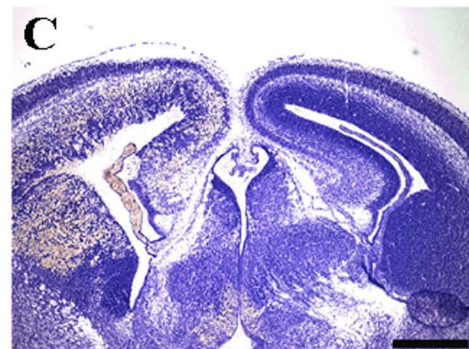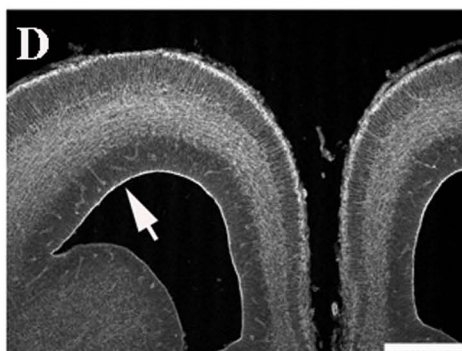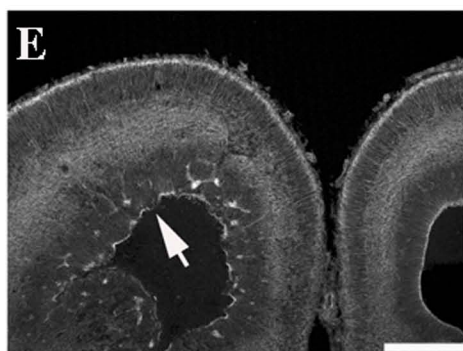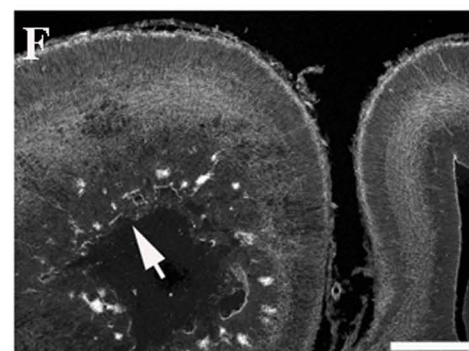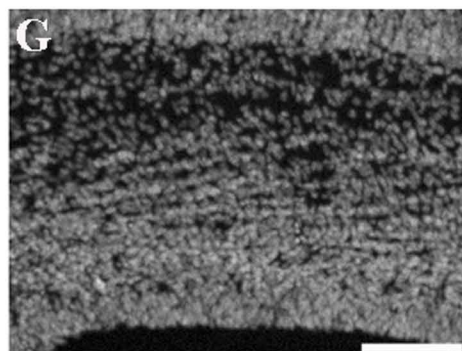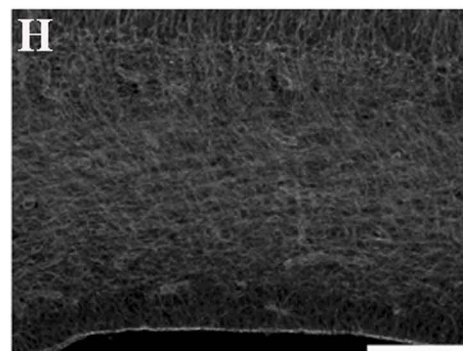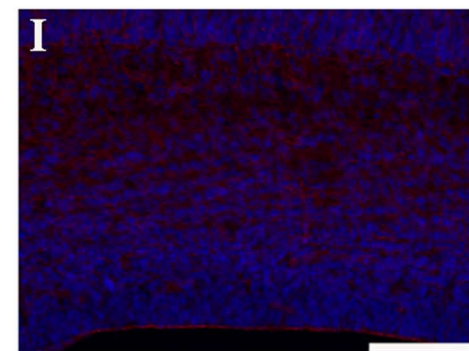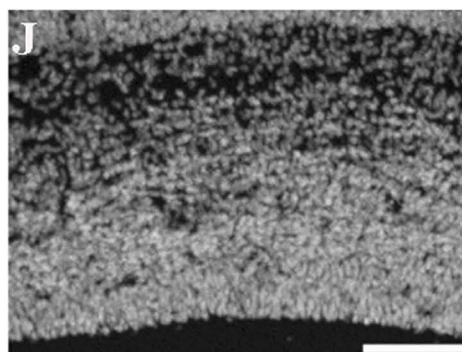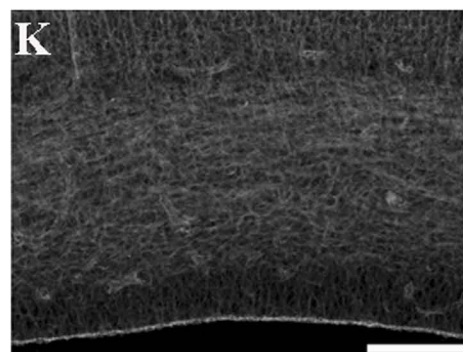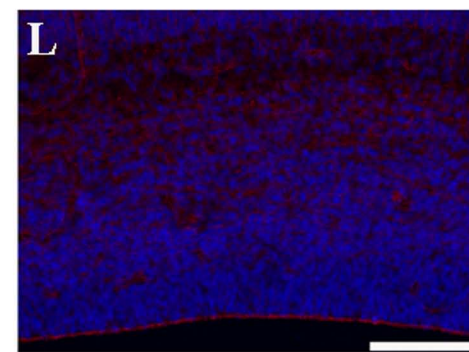

**Figure S16**

Supplement: Figure S16 — Effects of actin-perturbing drugs and Y-27632 on the apical actin filament of lateral ventricle wall. (A–F) Disruption of apical actin filament belt by intraventricular injection of cytochalasin D or latrunculin A. (A–C) Nissl staining of coronal brain sections of E15 embryos injected with control saline (A), or cytochalasin D (B) or latrunculin A (C). Embryos injected with cytochalasin D or latrunculin A showed disruption of the ventricular zone and swelling of the choroid plexus on the injected side. (A–C) Scale bars, 500 µm (A–C). (D–F) Phalloidin staining of coronal brain sections of control saline (D), cytochalasin D (E) and latrunculin A (F) injected embryos. Apical actin filament belt of the lateral wall was severely disrupted on the injected-side (arrows). (D–F) Scale bars, 250 µm. (G–L) Inhibition of ROCK by intraventricular injection of Y-27632 does not apparently affect cortical architecture. E15 wild-type embryos were intraventricularly injected with 0.5 µl of 100 µM Y-27632, a ROCK inhibitor (J, K, L) or saline as control (G, H, I). After 24 h, embryos were collected and analyzed. Coronal sections were stained with phalloidin (H, K and red in I, L) and Hoechst (G, J and blue in I, L). No obvious difference was observed between Y-27632 treated and control embryos. (G–L) Scale bars, 100 µm. (PDF) [file pone.0025465.s016.pdf]
