# Supplementary material for: Deficiency of mDia, an Actin Nucleator, Disrupts Integrity of Neuroepithelium and Causes Periventricular Dysplasia
Source: PLoS One. 2011 Sep 28;6(9):e25465. doi: 10.1371/journal.pone.0025465 (PMC3182227; doi:10.1371/journal.pone.0025465)
Supplement: Figure S6 — mDia depletion by RNAi disrupts apical actin filament and neuroepithelium integrity similarly to mDia-DKO mice. (A) NIH 3T3 cells were electroporated with plasmids encoding scramble shRNA (lane 1), shRNA's for mDia1 (lane 2), mDia2 (lane 3) or mDia3 (lane 4). Cells were lysed 72 h after electroporation and subjected to Western blotting for mDia1, mDia2, mDia3 and α-tubulin. Endogenous mDia1, mDia2 and mDia3 level were reduced after electroporation with the corresponding shRNA. α-tubulin was used as an internal control. (B) Coronal sections of the lateral ventricle wall at 72 h after electroporation with the plasmid encoding control scramble shRNA or mDia1/2/3 shRNA. EGFP was simultaneously introduced with shRNA to visualize transfected cells. mDia1/2/3 shRNA disrupted apical-basal polarity in neuroepithelial cells. Insets show higher magnification. Scale bar, 100 µm. (C) Phalloidin staining of coronal sections. mDia1/2/3 shRNA significantly reduced the fluorescent signal of the actin filament belt at the apical surface of the ventricular zone. Insets show higher magnification of the apical surface. (B, C) Scale bars, 100 µm. (PDF) [file pone.0025465.s006.pdf]

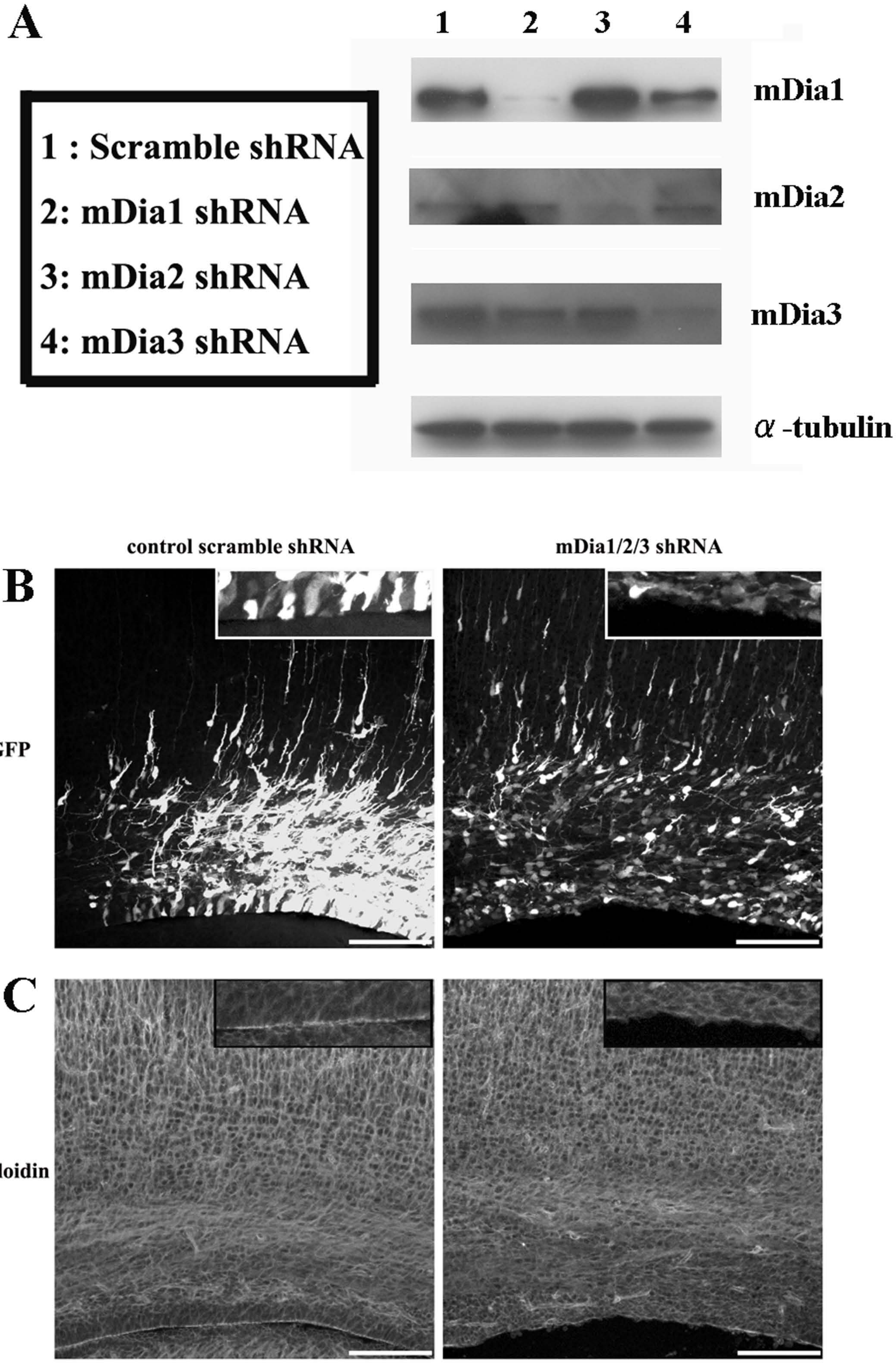
A
1
2
3
4
1 : Scramble shRNA
2: mDia1 shRNA
3: mDia2 shRNA
4: mDia3 shRNA
mDia1
mDia2
mDia3
α -tubulin
control scramble shRNA
mDia1/2/3 shRNA
B
EGFP
C
Phalloidin

Figure S6
