# Supplementary material for: Deficiency of mDia, an Actin Nucleator, Disrupts Integrity of Neuroepithelium and Causes Periventricular Dysplasia
Source: PLoS One. 2011 Sep 28;6(9):e25465. doi: 10.1371/journal.pone.0025465 (PMC3182227; doi:10.1371/journal.pone.0025465)
Supplement: Table S1 — Summary of microarray analysis on gene expression increased in E16 forebrain of mDia-DKO (n = 4) compared to mDia3null (n = 2). The probe sets up-regulated more than 1.5-fold are listed. (PDF) [file pone.0025465.s017.pdf]

Table S1

Name	RefSeq	description	average
Mllt3	NM_029931	myeloid/lymphoid or mixed lineage-leukemia translocation to 3 homolog (Drosophila) [Source:MarkerSymbol;Acc:MGI:1917372]	7.0
Hbb-y	NM_008221	hemoglobin Y, beta-like embryonic chain [Source:MarkerSymbol;Acc:MGI:96027]	6.6
LOC630212	XM_135925	–	4.8
A830080D0	XM_356366	RIKEN cDNA A830080D01 gene [Source:MarkerSymbol;Acc:MGI:2685992]	4.1
Hmgb1l	–	high mobility group box 1-like [Source:MarkerSymbol;Acc:MGI:3054046]	4.0
Ppfia2	NM_177373	protein tyrosine phosphatase, receptor type, f polypeptide (PTPRF), interacting protein (liprin), alpha 2 [Source:MarkerSymbol;Acc:MGI:24438	4.0
EG385412	XM_358228	–	3.8
Eif3s1	–	eukaryotic translation initiation factor 3, subunit 1 alpha [Source:MarkerSymbol;Acc:MGI:1925905]	3.5
Hba-x	NM_010405	hemoglobin X, alpha-like embryonic chain in Hba complex [Source:MarkerSymbol;Acc:MGI:96019]	3.5
1700073E1	NM_001001	RIKEN cDNA 1700073E17 gene [Source:MarkerSymbol;Acc:MGI:1920734]	3.5
EG668411	XM_357734	similar to 60S ribosomal protein L23a (LOC673782), mRNA [Source:RefSeq_dna;Acc:XR_004345]	3.4
Sumo2	XM_357457	SMT3 suppressor of mif two 3 homolog 2 (yeast) [Source:MarkerSymbol;Acc:MGI:2158813]	3.2
Kif15	NM_010620	kinesin family member 15 [Source:MarkerSymbol;Acc:MGI:1098258]	3.2
Nola2	NM_026631	nucleolar protein family A, member 2 [Source:MarkerSymbol;Acc:MGI:1098547]	3.0
EG668411	XM_195264	similar to 60S ribosomal protein L23a (LOC673782), mRNA [Source:RefSeq_dna;Acc:XR_004345]	3.0
Olfr345	NM_146945	olfactory receptor 345 [Source:MarkerSymbol;Acc:MGI:3030179]	3.0
Cdkl3	–	cyclin-dependent kinase-like 3 [Source:MarkerSymbol;Acc:MGI:2388268]	2.9
Fbln2	NM_007992	fibulin 2 [Source:MarkerSymbol;Acc:MGI:95488]	2.8
Aprin	NM_175310	androgen-induced proliferation inhibitor [Source:MarkerSymbol;Acc:MGI:2140945]	2.8
Lrrtm4	NM_178731	leucine rich repeat transmembrane neuronal 4 [Source:MarkerSymbol;Acc:MGI:2389180]	2.8
Ints6	NM_008715	integrator complex subunit 6 [Source:MarkerSymbol;Acc:MGI:1202397]	2.8
Gbp6	NM_145545	guanylate binding protein 6 [Source:MarkerSymbol;Acc:MGI:2444421]	2.7
Pmp2	XM_485204	peripheral myelin protein 2 [Source:MarkerSymbol;Acc:MGI:102667]	2.7
Esf1	XM_130548	ESF1, nucleolar pre-rRNA processing protein, homolog (S. cerevisiae) [Source:MarkerSymbol;Acc:MGI:1913830]	2.7
Cenpe	NM_173762	centromere protein E [Source:MarkerSymbol;Acc:MGI:1098230]	2.7
Eif3s6	NM_008388	eukaryotic translation initiation factor 3, subunit 6 [Source:MarkerSymbol;Acc:MGI:99257]	2.6
Tac1	–	tachykinin 1 [Source:MarkerSymbol;Acc:MGI:98474]	2.6
–	–	similar to ATP-binding cassette sub-family E member 1 (RNase L inhibitor) (Ribonuclease 4 inhibitor) (RNS4I) (LOC671837)	2.6
AW146154	–	expressed sequence AW146154 [Source:MarkerSymbol;Acc:MGI:2142212]	2.6
Wasl	XM_133026	Wiskott-Aldrich syndrome-like (human) [Source:MarkerSymbol;Acc:MGI:1920428]	2.5
4933411G0	–	RIKEN cDNA 4933411G06 gene [Source:MarkerSymbol;Acc:MGI:1918340]	2.5
BC002059	NM_145397	cDNA sequence BC002059 [Source:MarkerSymbol;Acc:MGI:2384864]	2.4
Sucla2	NM_011506	succinate-Coenzyme A ligase, ADP-forming, beta subunit [Source:MarkerSymbol;Acc:MGI:1306775]	2.4
Nexn	NM_199465	nexilin [Source:MarkerSymbol;Acc:MGI:1916060]	2.4
Tmem67	NM_177861	transmembrane protein 67 [Source:MarkerSymbol;Acc:MGI:1923928]	2.4
Tmem16b	NM_153589	transmembrane protein 16B [Source:MarkerSymbol;Acc:MGI:2387214]	2.3
Trhde	NM_146241	TRH-degrading enzyme [Source:MarkerSymbol;Acc:MGI:2384311]	2.3
–	XM_205529	similar to MAPKK-like protein kinase (LOC621880), mRNA [Source:RefSeq_dna;Acc:XR_003283]	2.3
NP_001019	–	similar to zinc finger protein 97 (LOC635426), mRNA [Source:RefSeq_dna;Acc:XR_004307]	2.3
Cdca8	NM_026560	cell division cycle associated 8 [Source:MarkerSymbol;Acc:MGI:1196274]	2.3
Hisppd1	NM_173760	histidine acid phosphatase domain containing 1 [Source:MarkerSymbol;Acc:MGI:2142810]	2.3
Clic3	NM_027085	chloride intracellular channel 3 [Source:MarkerSymbol;Acc:MGI:1916704]	2.3
Yaf2	NM_024189	YY1 associated factor 2 [Source:MarkerSymbol;Acc:MGI:1914307]	2.3
Calu	NM_184053	calumenin [Source:MarkerSymbol;Acc:MGI:1097158]	2.3
EG225058	XM_140116	predicted gene, EG225058 [Source:MarkerSymbol;Acc:MGI:3648524]	2.3
NP_001013	–	similar to Eukaryotic translation initiation factor 1A (eIF-1A) (eIF-4C) (LOC435337), mRNA [Source:RefSeq_dna;Acc:NM_001013824]	2.2
Rabep1	NM_019400	rabaptin, RAB GTPase binding effector protein 1 [Source:MarkerSymbol;Acc:MGI:1860236]	2.2
2310001H1	NM_145563	RIKEN cDNA 2310001H12 gene [Source:MarkerSymbol;Acc:MGI:1916754]	2.2
Zfp708	XM_484273	zinc finger protein 708 [Source:MarkerSymbol;Acc:MGI:3040674]	2.2
Pstk	NM_177609	phosphoseryl-tRNA kinase [Source:MarkerSymbol;Acc:MGI:2685945]	2.2
Myo9a	XM_356161	myosin IXa [Source:MarkerSymbol;Acc:MGI:107735]	2.1
Hes1	NM_008235	hairy and enhancer of split 1 (Drosophila) [Source:MarkerSymbol;Acc:MGI:104853]	2.1
2310001H1	–	RIKEN cDNA 2310001H12 gene [Source:MarkerSymbol;Acc:MGI:1916754]	2.1
Aga	NM_001005	aspartylglucosaminidase [Source:MarkerSymbol;Acc:MGI:104873]	2.1
2410141K0	NM_183119	RIKEN cDNA 2410141K09 gene [Source:MarkerSymbol;Acc:MGI:1924053]	2.1
Gstcd	–	glutathione S-transferase, C-terminal domain containing [Source:MarkerSymbol;Acc:MGI:1914803]	2.1
Olfr147	NM_146869	olfactory receptor 147 [Source:MarkerSymbol;Acc:MGI:2660712]	2.1
Ifit2	NM_008332	interferon-induced protein with tetratricopeptide repeats 2 [Source:MarkerSymbol;Acc:MGI:99449]	2.1
2510049I19	XM_357740	RIKEN cDNA 2510049I19 gene [Source:MarkerSymbol;Acc:MGI:1915172]	2.1
–	–	similar to ribosomal protein S15a (LOC435784), mRNA [Source:RefSeq_dna;Acc:XR_002799]	2.1
A530013C2	NM_177851	RIKEN cDNA A530013C23 gene [Source:MarkerSymbol;Acc:MGI:3041178]	2.1
2210409E1	NM_028218	RIKEN cDNA 2210409E12 gene [Source:MarkerSymbol;Acc:MGI:1919631]	2.1
Bmi1	NM_007552	B lymphoma Mo-MLV insertion region 1 [Source:MarkerSymbol;Acc:MGI:88174]	2.0
C330022B2	–	RIKEN cDNA C330022B21 gene [Source:MarkerSymbol;Acc:MGI:1925949]	2.0
Smek1	NM_211355	SMEK homolog 1, suppressor of mek1 (Dictyostelium) [Source:MarkerSymbol;Acc:MGI:1915984]	2.0
Mysm1	NM_177239	myb-like, SWIRM and MPN domains 1 [Source:MarkerSymbol;Acc:MGI:2444584]	2.0
Nrk	NM_013724	Nik related kinase [Source:MarkerSymbol;Acc:MGI:1351326]	2.0
Hscb	NM_153571	HscB iron-sulfur cluster co-chaperone homolog (E. coli) [Source:MarkerSymbol;Acc:MGI:2141135]	2.0
2310047C0	XM_358866	RIKEN cDNA 2310047C04 gene [Source:MarkerSymbol;Acc:MGI:1917433]	2.0
AW456874	NM_207232	expressed sequence AW456874 [Source:MarkerSymbol;Acc:MGI:2145430]	2.0
Meis1	–	myeloid ecotropic viral integration site 1 [Source:MarkerSymbol;Acc:MGI:104717]	2.0
Tec	NM_013689	cytoplasmic tyrosine kinase, Dscr28C related (Drosophila) [Source:MarkerSymbol;Acc:MGI:98662]	2.0
Phf14	NM_029404	PHD finger protein 14 [Source:MarkerSymbol;Acc:MGI:1923539]	2.0
Tchp	NM_029992	trichoplein, keratin filament binding [Source:MarkerSymbol;Acc:MGI:1925082]	2.0
2310003C2	XM_356730	RIKEN cDNA 2310003C23 gene [Source:MarkerSymbol;Acc:MGI:1923675]	2.0

Ncapg	XM_485604	on-SMC condensin I complex, subunit G [Source:MarkerSymbol;Acc:MGI:1930197]	2.0
Ccdc72	–	coiled-coil domain containing 72 [Source:MarkerSymbol;Acc:MGI:1913417]	2.0
Atp13a4	NM_172613	ATPase type 13A4 [Source:MarkerSymbol;Acc:MGI:1924456]	2.0
Alg13	–	asparagine-linked glycosylation 13 homolog (S. cerevisiae) [Source:MarkerSymbol;Acc:MGI:1914824]	2.0
Irak4	NM_029926	interleukin-1 receptor-associated kinase 4 [Source:MarkerSymbol;Acc:MGI:2182474]	2.0
Zfp97	NM_011765	zinc finger protein 97 [Source:MarkerSymbol;Acc:MGI:105921]	1.9
Zfp560	NM_001004	zinc finger protein 560 [Source:MarkerSymbol;Acc:MGI:1915280]	1.9
Wdr44	XM_135805	WD repeat domain 44 [Source:MarkerSymbol;Acc:MGI:1919654]	1.9
D830030K2	NM_177135	RIKEN cDNA D830030K20 gene [Source:MarkerSymbol;Acc:MGI:2443830]	1.9
–	NM_001002	ribosomal protein L17 (Rpl17), mRNA [Source:RefSeq_dna;Acc:NM_001002239]	1.9
Ndufb4	NM_026610	NADH dehydrogenase (ubiquinone) 1 beta subcomplex 4 [Source:MarkerSymbol;Acc:MGI:1915444]	1.9
Atxn7l3	–	ataxin 7-like 3 [Source:MarkerSymbol;Acc:MGI:3036270]	1.9
Zfp160	NM_145483	zinc finger protein 160 [Source:MarkerSymbol;Acc:MGI:108187]	1.9
Emb	NM_010330	embigin [Source:MarkerSymbol;Acc:MGI:95321]	1.9
A430106J1	–	RIKEN cDNA A430106J12 gene [Source:MarkerSymbol;Acc:MGI:1925177]	1.9
BC032265	NM_181420	cDNA sequence BC032265 [Source:MarkerSymbol;Acc:MGI:2679256]	1.9
Zfp618	XM_143826	zinc fingerprotein 618 [Source:MarkerSymbol;Acc:MGI:1919950]	1.9
5730577I03	NM_175110	RIKEN cDNA 5730577I03 gene [Source:MarkerSymbol;Acc:MGI:1913912]	1.9
C1d	NM_020558	nuclear DNA binding protein [Source:MarkerSymbol;Acc:MGI:1927354]	1.9
D030022P0	XM_486008	RIKEN cDNA D030022P06 gene [Source:MarkerSymbol;Acc:MGI:2444036]	1.9
4932438A1	–	RIKEN cDNA 4932438A13 gene [Source:MarkerSymbol;Acc:MGI:2444631]	1.9
Snx6	NM_026998	sorting nexin 6 [Source:MarkerSymbol;Acc:MGI:1919433]	1.9
BC031441	NM_146249	cDNA sequence BC031441 [Source:MarkerSymbol;Acc:MGI:2385323]	1.8
Upf3b	XM_110787	UPF3 regulator of nonsense transcripts homolog B (yeast) [Source:MarkerSymbol;Acc:MGI:1915384]	1.8
Amd1	NM_007444	S-adenosylmethionine decarboxylase 1 [Source:MarkerSymbol;Acc:MGI:88004]	1.8
Hist1h2aa	NM_175658	histone cluster 1, H2aa [Source:MarkerSymbol;Acc:MGI:2448285]	1.8
0610013E2	NM_029788	RIKEN cDNA 0610013E23 gene [Source:MarkerSymbol;Acc:MGI:1924142]	1.8
Ddx55	NM_026409	DEAD (Asp-Glu-Ala-Asp) box polypeptide 55 [Source:MarkerSymbol;Acc:MGI:1915098]	1.8
Ankhd1	–	ankyrin repeat and KH domain containing 1 [Source:MarkerSymbol;Acc:MGI:1921733]	1.8
Esf1	XM_130548	ESF1, nucleolar pre-rRNA processing protein, homolog (S. cerevisiae) [Source:MarkerSymbol;Acc:MGI:1913830]	1.8
3110003A1	XM_125510	RIKEN cDNA 3110003A17 gene [Source:MarkerSymbol;Acc:MGI:1920362]	1.8
Dnajc10	NM_024181	DnaJ (Hsp40) homolog, subfamily C, member 10 [Source:MarkerSymbol;Acc:MGI:1914111]	1.8
Scye1	NM_007926	small inducible cytokine subfamily E, member 1 [Source:MarkerSymbol;Acc:MGI:102774]	1.8
Exosc3	NM_025513	exosome component 3 [Source:MarkerSymbol;Acc:MGI:1913612]	1.8
LOC640480	XM_357154	–	1.8
–	–	similar to Poly(rC)-binding protein 2 (Alpha-CP2) (Putative heterogeneous nuclear ribonucleoprotein X) (hnRNP X) (CTBP) (CBP) (LOC66721	1.8
Ccar1	NM_026201	cell division cycle and apoptosis regulator 1 [Source:MarkerSymbol;Acc:MGI:1914750]	1.8
Cetn3	NM_007684	centrin 3 [Source:MarkerSymbol;Acc:MGI:1097706]	1.8
A430107O1	XM_145117	RIKEN cDNA A430107O13 gene [Source:MarkerSymbol;Acc:MGI:2444814]	1.8
–	NM_009446	tubulin, alpha 3 (Tuba3), mRNA [Source:RefSeq_dna;Acc:NM_009446]	1.8
Surb7	NM_025315	SRB7 (suppressor of RNA polymerase B) homolog (S. cerevisiae) [Source:MarkerSymbol;Acc:MGI:1347064]	1.8
39332	NM_009859	septin 7 [Source:MarkerSymbol;Acc:MGI:1335094]	1.8
Sfpq	NM_023603	splicing factor proline/glutamine rich (polypyrimidine tract binding protein associated) [Source:MarkerSymbol;Acc:MGI:1918764]	1.8
Pik4ca	NM_001001	phosphatidylinositol 4-kinase, catalytic, alpha polypeptide [Source:MarkerSymbol;Acc:MGI:2448506]	1.7
Stim2	XM_132038	stromal interaction molecule 2 [Source:MarkerSymbol;Acc:MGI:2151156]	1.7
Pnn	NM_008891	pinin [Source:MarkerSymbol;Acc:MGI:1100514]	1.7
Eif2s1	NM_026114	eukaryotic translation initiation factor 2, subunit 1 alpha [Source:MarkerSymbol;Acc:MGI:95299]	1.7
Wapal	NM_001004	wings apart-like homolog (Drosophila) [Source:MarkerSymbol;Acc:MGI:2675859]	1.7
Matr3	NM_010771	matrin 3 [Source:MarkerSymbol;Acc:MGI:1298379]	1.7
Cenpc1	NM_007683	centromere protein C1 [Source:MarkerSymbol;Acc:MGI:99700]	1.7
Cxxc5	NM_133687	CXXC finger 5 [Source:MarkerSymbol;Acc:MGI:1914643]	1.7
Zfp40	NM_009555	zinc finger protein 40 [Source:MarkerSymbol;Acc:MGI:99185]	1.7
Kcnk18	NM_207261	potassium channel, subfamily K, member 18 [Source:MarkerSymbol;Acc:MGI:2685627]	1.7
Fmr1	NM_008031	fragile X mental retardation syndrome 1 homolog [Source:MarkerSymbol;Acc:MGI:95564]	1.7
Styx	–	phosphoserine/threonine/tyrosine interaction protein [Source:MarkerSymbol;Acc:MGI:1891150]	1.7
Fancm	NM_178912	Fanconi anemia, complementation group M [Source:MarkerSymbol;Acc:MGI:2442306]	1.7
Trio	–	triple functional domain (PTPRF interacting) [Source:MarkerSymbol;Acc:MGI:1927230]	1.7
Prkrir	NM_028410	protein-kinase, interferon-inducible double stranded RNA dependent inhibitor, repressor of (P58 repressor) [Source:MarkerSymbol;Acc:MGI:1	1.7
EG436332	–	predicted gene, EG436332 [Source:MarkerSymbol;Acc:MGI:3643508]	1.7
Nuf2	NM_023284	NUF2, NDC80 kinetochore complex component, homolog (S. cerevisiae) [Source:MarkerSymbol;Acc:MGI:1914227]	1.7
Npm1	–	nucleophosmin 1 [Source:MarkerSymbol;Acc:MGI:106184]	1.7
Rps11	NM_013725	ribosomal protein S11 [Source:MarkerSymbol;Acc:MGI:1351329]	1.7
Nrip1	NM_173440	nuclear receptor interacting protein 1 [Source:MarkerSymbol;Acc:MGI:1315213]	1.7
5730507C0	–	RIKEN cDNA 5730507C01 gene [Source:MarkerSymbol;Acc:MGI:1917882]	1.7
Polr3k	NM_025901	polymerase (RNA) III (DNA directed) polypeptide K [Source:MarkerSymbol;Acc:MGI:1914255]	1.6
Tmem97	NM_133706	transmembrane protein 97 [Source:MarkerSymbol;Acc:MGI:1916321]	1.6
Wdhd1	NM_172598	WD repeat and HMG-box DNA binding protein 1 [Source:MarkerSymbol;Acc:MGI:2443514]	1.6
Fkbp3	–	FK506 binding protein 3 [Source:MarkerSymbol;Acc:MGI:1353460]	1.6
Dhx15	–	DEAH (Asp-Glu-Ala-His) box polypeptide 15 [Source:MarkerSymbol;Acc:MGI:1099786]	1.6
Amy1	NM_007446	amylase 1, salivary [Source:MarkerSymbol;Acc:MGI:88019]	1.6
Usp1	NM_146144	ubiquitin specific peptdiase 1 [Source:MarkerSymbol;Acc:MGI:2385198]	1.6
Hist1h2bg	–	histone cluster 1, H2bg [Source:MarkerSymbol;Acc:MGI:2448386]	1.6
Ncor1	NM_011308	nuclear receptor co-repressor 1 [Source:MarkerSymbol;Acc:MGI:1349717]	1.6
Nap1l3	NM_138742	nucleosome assembly protein 1-like 3 [Source:MarkerSymbol;Acc:MGI:1859565]	1.6
BC016423	NM_134063	cDNA sequence BC016423 [Source:MarkerSymbol;Acc:MGI:2145274]	1.6
Lsm7	NM_025349	LSM7 homolog, U6 small nuclear RNA associated (S. cerevisiae) [Source:MarkerSymbol;Acc:MGI:1913344]	1.6
Ctsl	NM_009984	cathepsin L [Source:MarkerSymbol;Acc:MGI:88564]	1.6
Slc25a3	NM_133668	solute carrier family 25 (mitochondrial carrier, phosphate carrier), member 3 [Source:MarkerSymbol;Acc:MGI:1353498]	1.6
Dnajc15	NM_025384	DnaJ (Hsp40) homolog, subfamily C, member 15 [Source:MarkerSymbol;Acc:MGI:1913398]	1.6
Txndc1	NM_028339	thioredoxin domain containing 1 [Source:MarkerSymbol;Acc:MGI:1919986]	1.6
D2Ertd435e	NM_153387	Gamma-tubulin complex component 4 (GCP-4). [Source:Uniprot/SWISSPROT;Acc:Q9D4F8]	1.6
Fut9	NM_010243	fucosyltransferase 9 [Source:MarkerSymbol;Acc:MGI:1330859]	1.6
Ppp2r5e	NM_012024	protein phosphatase 2, regulatory subunit B (B56), epsilon isoform [Source:MarkerSymbol;Acc:MGI:1349473]	1.5
Hmgcr	–	3-hydroxy-3-methylglutaryl-Coenzyme A reductase [Source:MarkerSymbol;Acc:MGI:96159]	1.5
