# Supplementary material for: Deficiency of mDia, an Actin Nucleator, Disrupts Integrity of Neuroepithelium and Causes Periventricular Dysplasia
Source: PLoS One. 2011 Sep 28;6(9):e25465. doi: 10.1371/journal.pone.0025465 (PMC3182227; doi:10.1371/journal.pone.0025465)
Supplement: Table S2 — Summary of microarray analysis of on gene expression decreased in E16 forebrain of mDia-DKO (n = 4) compared to mDia3null (n = 2). The probe sets down-regulated less than 0.7-fold are listed. (PDF) [file pone.0025465.s018.pdf]

Table S2

Name	RefSeq	description	average
Diap1	NM_007858	diaphanous homolog 1 (Drosophila) [Source:MarkerSymbol;Acc:MGI:1194490]	0.31
Hba-a1	NM_008218	hemoglobin alpha, adult chain 1 [Source:MarkerSymbol;Acc:MGI:96015]	0.44
Crybb1	NM_023695	crystallin, beta B1 [Source:MarkerSymbol;Acc:MGI:104992]	0.49
Rpl41	NM_018860	ribosomal protein L41 [Source:MarkerSymbol;Acc:MGI:1915195]	0.53
Serpinf2	NM_008878	serine (or cysteine) peptidase inhibitor, clade F, member 2 [Source:MarkerSymbol;Acc:MGI:107173]	0.54
Ubb	NM_011664	ubiquitin B [Source:MarkerSymbol;Acc:MGI:98888]	0.54
Ubb	XM_122700	ubiquitin B [Source:MarkerSymbol;Acc:MGI:98888]	0.54
Actg1	NM_009609	actin, gamma, cytoplasmic 1 [Source:MarkerSymbol;Acc:MGI:87906]	0.55
Eef1a1	NM_010106	eukaryotic translation elongation factor 1 alpha 1 [Source:MarkerSymbol;Acc:MGI:1096881]	0.59
Tubb5	NM_011655	tubulin, beta 5 [Source:MarkerSymbol;Acc:MGI:107812]	0.59
Hdac3	NM_010411	histone deacetylase 3 [Source:MarkerSymbol;Acc:MGI:1343091]	0.60
Dppa3	NM_139218	developmental pluripotency-associated 3 [Source:MarkerSymbol;Acc:MGI:1920958]	0.60
Rps3	NM_012052	ribosomal protein S3 [Source:MarkerSymbol;Acc:MGI:1350917]	0.60
Sectm1b	NM_026907	secreted and transmembrane 1B [Source:MarkerSymbol;Acc:MGI:1929083]	0.61
Olfr77	NM_146339	olfactory receptor 77 [Source:MarkerSymbol;Acc:MGI:2153206]	0.61
Mast3	-	microtubule associated serine/threonine kinase 3 [Source:MarkerSymbol;Acc:MGI:2683541]	0.62
Rps2	NM_008503	ribosomal protein S2 [Source:MarkerSymbol;Acc:MGI:105110]	0.63
Slc5a1	NM_019810	solute carrier family 5 (sodium/glucose cotransporter), member 1 [Source:MarkerSymbol;Acc:MGI:107678]	0.64
Tubb2a	-	tubulin, beta 2a [Source:MarkerSymbol;Acc:MGI:107861]	0.65
